# iDrug: Integration of drug repositioning and drug-target prediction via cross-network embedding

**DOI:** 10.1371/journal.pcbi.1008040

**Published:** 2020-07-15

**Authors:** Huiyuan Chen, Feixiong Cheng, Jing Li

**Affiliations:** 1 Department of Computer and Data Sciences, Case Western Reserve University, Cleveland, Ohio, United States of America; 2 Genomic Medicine Institute, Lerner Research Institute, Cleveland Clinic, Cleveland, Ohio, United States of America; 3 Department of Molecular Medicine, Cleveland Clinic Lerner College of Medicine, Case Western Reserve University, Cleveland, Ohio, United States of America; 4 Case Comprehensive Cancer Center, Case Western Reserve University School of Medicine, Cleveland, Ohio, United States of America; Icahn School of Medicine at Mount Sinai, UNITED STATES

## Abstract

Computational drug repositioning and drug-target prediction have become essential tasks in the early stage of drug discovery. In previous studies, these two tasks have often been considered separately. However, the entities studied in these two tasks (i.e., drugs, targets, and diseases) are inherently related. On one hand, drugs interact with targets in cells to modulate target activities, which in turn alter biological pathways to promote healthy functions and to treat diseases. On the other hand, both drug repositioning and drug-target prediction involve the same drug feature space, which naturally connects these two problems and the two domains (diseases and targets). By using the *wisdom of the crowds*, it is possible to transfer knowledge from one of the domains to the other. The existence of relationships among drug-target-disease motivates us to jointly consider drug repositioning and drug-target prediction in drug discovery. In this paper, we present a novel approach called iDrug, which seamlessly integrates drug repositioning and drug-target prediction into one coherent model via cross-network embedding. In particular, we provide a principled way to transfer knowledge from these two domains and to enhance prediction performance for both tasks. Using real-world datasets, we demonstrate that iDrug achieves superior performance on both learning tasks compared to several state-of-the-art approaches. Our code and datasets are available at: https://github.com/Case-esaC/iDrug.

## Author summary

Traditional high-throughput techniques for drug discovery are often expensive, time-consuming, and with high failure rates. Computational drug repositioning and drug-target prediction have thus become essential tasks in the early stage drug discovery. The emergence of large-scale heterogeneous biological networks has offered unprecedented opportunities for developing machine learning approaches to identify novel drug-disease or drug-target interactions. However, most existing works focused either on the drug-disease network or on the drug-target network, thus failed to capture the inherent dependencies between these two networks. These two biological networks are naturally connected since they involve the same drug feature space. In our opinion, ignoring this rich source of information is a major shortcoming of some existing works. In this paper, we present a novel approach called iDrug, which seamlessly integrates the drug-disease network and the drug-target network into one coherent model via cross-network embedding. As a result, iDrug is able to take full usage of the knowledge within these two biological networks to better exploit new biomedical insights of drug-target-disease. Therefore, iDrug has broad applications in drug discovery.

This is a *PLOS Computational Biology* Methods paper.

## Introduction

Targeted therapies and personalized treatments are the most promising strategies to treat complex human diseases, especially for cancer. Accurate identification of drug mechanism of actions (MoAs) is thus of great importance in drug discovery process. Two important tasks, drug repositioning (also known as drug-disease prediction) [1] and drug-target prediction [2], have been actively investigated to better understand the drugs’ MoAs. Drug repositioning (as well as drug-target prediction), aiming to identify new indications (new targets) for existing drugs, had gained increasing interests over the last decades. Traditional *in vitro* and *in vivo* prediction of interactions between drug-disease or drug-target are desirable in many cases, but with an expensive and protracted process of experimentation and testing [3, 4]. On the other hand, computational approaches provide an alternative tool to efficiently predict potential candidates with certain reasonable accuracy, thus narrowing down the search space to be investigated by the follow-up wet-lab experiments [5].

Many computational approaches have been developed for each task independently. Two excellent surveys on drug repositioning [6] and drug-target prediction [7] contain a very detailed overview of different machine learning techniques for each domain. Among many different methods, one of the most popular approaches is the network-based inference model [8, 9], which formulates drug-disease (or drug-target) prediction as a missing link prediction problem on a heterogeneous network. Advances in this direction are also essential for identifying biological significance of new disease genes, and for uncovering drug targets and biomarkers for complex diseases.

For drug repositioning, Wu et al. proposed a weighted bipartite network to identify the connected communities of drugs and diseases using a network clustering approach [10]. Two network topology-based methods, ProbS and HeatS, were also introduced to predict new indications for different diseases by considering the disease pathway and phenotype features [11]. MBiRW further used a bi-random walk algorithm on a two-layer network to identify potential novel indications for a given drug [12]. Zhang et al. introduced a matrix factorization method to predict novel drug-disease associations by integrating multiple drug and disease similarities [13]. Similarly, Chen et al. further applied multiple kernel learning to incorporate multiple heterogeneous data sources of drug and disease into the prediction framework [14]. Recently, Zeng et al. introduced a network-based deep learning method for *in silico* drug repositioning, which could learn nonlinear features of drugs from the heterogeneous networks by a multi-modal deep autoencoder [15].

For drug-target prediction, Bleakley et al. applied support vector machine to predict novel targets based on a bipartite local model [16]. Chen et al. presented a random walk with restart on a bipartite network to predict potential drug-target interactions on a large scale [17]. Ezzat et al. proposed two matrix factorization methods for drug-target prediction and constantly boost the accuracy via graph regularization. In addition, Zheng et al. proposed a coupled matrix factorization model, which projected drugs and targets into a common low-rank feature space [18]. Nascimento et al. also integrated multiple heterogeneous information sources for both drugs and targets by using multiple kernel learning [19]. Chen et al. developed several effective computational models to predict potential drug–target interactions from heterogeneous biological data, which could provide better understanding of various interactions [20]. Luo et al. presented a network integration pipeline for drug-target prediction via low-rank matrix factorization, which integrated diverse information from heterogeneous data sources [21]. Recently, Lee et al. constructed a novel drug-target prediction model to extract local residue patterns of target protein sequences using a CNN-based deep learning approach, which exhibited better performance than previous shallow models [22].

Computational frameworks from the two domains share many common characteristics. First, most of them, for both domains, obey the *guilt by association* principle, which assumes that similar drugs tend to treat (bind to) similar diseases (targets) with high probability and vice versa [6, 7]. Second, they have exactly the same network structures, i.e., a bipartite network consisting of one layer of drugs and the other layer of diseases (targets). Moreover, they adopt many common features of drugs, targets, and diseases, such as drug’s chemical structure, target’s sequence, and disease phenotype. Based on those commonalities, it is not surprising that same machine learning methods can be adopted or directly applied to two prediction tasks interchangeably with barely no compromise on accuracy. For example, random walk with restart [17], couple matrix factorization [18], and multiple kernel learning [19] were first successfully proposed to predict the new drug-target interactions. Subsequently, they can be perfectly adaptive to solve drug repositioning problem as well [12–14].

It is generally a challenging task to compare different approaches in either domain for a couple of reasons. First, many methods are not only different in the computational approaches that they used, most of the time, they are also very different in the data that they analyzed. Sometimes, it is just impractical to separate data from computational approaches. Second, many of the approaches are based on the global structure of the network in predicting missing links. Although they normally give better results comparing to methods based on local structure, they may not provide intuitive explanations of the predicted results. Third, most computational approaches cannot afford experimental validations. Coupled with the issue of different types of input data required, it is hard to compare different methods objectively. One possible solution for this problem is through crowdsourcing projects such as the Dream Challenges [23]. Regardless these challenges, there is still room to improve computational approaches. In particular, most of the existing studies considered drug-disease and drug-target prediction as two isolated tasks and the relationships between these two domains—namely, cross-domain knowledge—are typically ignored. Some recent studies have shown that such cross-domain knowledge is very useful in improving the success rate of drug development [24]. Indeed, therapeutic effect of drugs on a disease is through their abilities to modulate the biological targets within the disease pathways, which in turn promotes healthy functioning of the metabolic system and cure the disease. In other words, targets can provide evidence to understand drugs’ MoAs, which could serve as a useful bridge in drug discovery. Therefore, it is reasonable to integrate drug repositioning and drug-target prediction together to better exploit different domain-specific knowledge. Wang et al. presented a three-layer heterogeneous network model named TL_HGBI to learn the potential relationships among drug-target-disease in a unified model [25]. However, it can only be applied to a small-scale dataset since its drug layer required that the drugs must interact with known targets and diseases, leading to the issue of data sparsity. In real-life situation, due to the nature of data collection (i.e., data were generated by different labs in different time), it is very unlikely that the triple relationships <drug, target, disease> are always available while integrating drug-disease and drug-target domain. Moreover, the random walk with restart algorithm adopted by TL_HGBI assumed that the next step of the random walker only depended on the current node, which might suffer from the bias induced by noise. The limitation motivates us to seek answers to a natural question: can we still leverage all the available data provided in two domains to alleviate the data sparsity issue, generate better performance, and extend to large-scale dataset in drug discovery?

**Fig 1:**
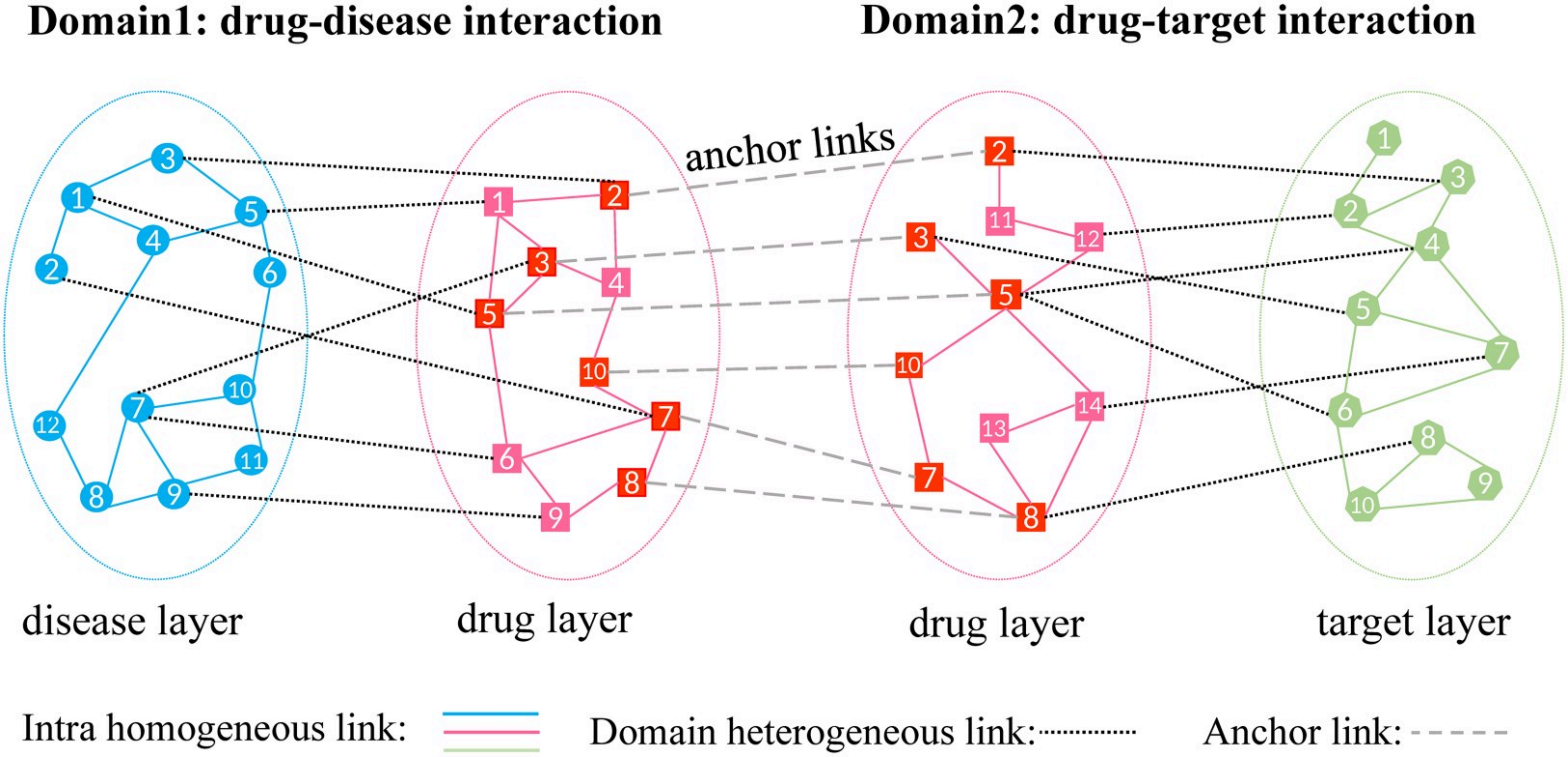
An overview of iDrug. An illustration of the cross-network framework across two domains: drug-disease and drug-target networks. iDrug requires only partially overlapped drug nodes between these two domains. The anchor links among drug nodes are used to transfer domain knowledge across the networks.

In this work, we propose a novel framework—iDrug, which not only jointly performs drug repositioning and drug-target prediction at the same time, but also integrates diverse information from heterogeneous data sources. The key idea of iDrug is mainly inspired by cross-network embedding [26–28], which aims to borrow information from some related domains to achieve better performance in the domain of interest. Compared with single network embedding, cross-network embedding simultaneously considers at least two types of networks from different domains [27]. To be specific, two types of relationships are considered for each node in the networks: (i) *within-network relationship*, which preserves the specific structural feature of a node in its own domain; (ii) *cross-network dependency*, which describes the associations between nodes across different networks/domains. Fig 1 shows an example of two heterogeneous networks corresponding to two domains $D_{1}$ and $D_{2}$. In each domain, the edges connecting those nodes in the same layer (e.g., the disease layer) are defined based on their similarities (e.g., disease-disease similarity). The edges across two different layers in the same domain (e.g., drug-disease links) are labeled based on known associations. The goal is to predict novel cross links in the same domain, which solves the drug repositioning or drug-target prediction problem. Note that several drug nodes such as {②, ③, ⑤, ⑦, ⑧, ⑩} in Fig 1 also have another type of links, which connects the same drug nodes between $D_{1}$ and $D_{2}$. We call these links as *anchor links*, which have been shown to play a central role in multi-layered network mining tasks [29, 30]. We thus regard these anchor links as bridges to fully transfer domain-specific knowledge to benefit each other during the learning process. iDrug has several advantages over existing single-domain methods. First, unlike single-domain approaches, iDrug is able to jointly perform two tasks, drug repositioning and drug-target prediction in one unified model, which has broader applicability in real-life drug discovery. In addition, by transferring knowledge across different domains, iDrug can substantially alleviate data sparsity issue due to complementary property of the two related domains and thus mutually enhance the performance. Moreover, unlike some previous methods that requires totally overlap of the drug layer [25, 31], iDrug only requires partial overlap of drugs from the two domains. Therefore, it will be able to include more data from both domains. Overall, iDrug provides an alternative opportunity for us to better gain new biomedical insights of drug-target-disease relationships.

## Materials and methods

In this section, we first formulate the drug repositioning and drug-target prediction problem as a matrix completion problem. We then provide the details of multiple heterogeneous data sources, the framework of iDrug, and the learning algorithm.

### Problem definition

#### Notation

Following the convention, we use bold upper-case for matrices (e.g., **A**), the (*i*, *j*)-th element of matrix **A** denotes as **A**(*i*, *j*). **A**(*i*,:) or **A**(:, *i*) denotes its *i*-th row or column and **A**^*T*^ denotes the transpose of matrix **A**.

#### Domain 1: Drug-disease prediction

In domain $D_{1}$, we try to predict new indications of drugs using a drug-disease bipartite network, drug-drug similarity, and disease-disease similarity information. We start by representing the bipartite network as a sparse *n*_1_ × *m*_1_ matrix **X**^(1)^, where *n*_1_ is the number of drugs and *m*_1_ is the number of diseases. **X**^(1)^(*i*, *j*) = 1 if *i*-th drug and *j*-th disease are known to interact and **X**^(1)^(*i*, *j*) = 0 otherwise. The drug-drug similarity can be encoded into a *n*_1_ × *n*_1_ square matrix $A_{u}^{\left(1\right)}$, with $A_{u}^{\left(1\right)}\left(i,j\right)$ representing the similarity score between *i*-th and *j*-th drugs. Analogously, the disease-disease similarity can be represented by a *m*_1_ × *m*_1_ square matrix $A_{v}^{\left(1\right)}$. We can then regard this problem as an analog of user-item preferences problem in recommender system [32], in which users and items denote drugs and diseases, respectively. The goal is to predict the new interactions between drugs and diseases by completing the matrix **X**^(1)^.

#### Domain 2: Drug-target prediction

Similarly, we denote the drug-target bipartite network as a sparse matrix $X^{\left(2\right)}\in R^{n_{2}\times m_{2}}$ in domain $D_{2}$, where *n*_2_ and *m*_2_ are the numbers of drugs and targets, respectively. The matrices $A_{u}^{\left(2\right)}\in R^{n_{2}\times n_{2}}$ and $A_{v}^{\left(2\right)}\in R^{m_{2}\times m_{2}}$ denote the drug-drug similarity and target-target similarity, respectively. Note that both $A_{u}^{\left(1\right)}$ and $A_{u}^{\left(2\right)}$ represent drug-drug similarity but in different domains, resulting in different sizes. Here we only require partial overlap of drugs between $D_{1}$ and $D_{2}$. Novel drug-target interactions can then be inferred by completing the matrix **X**^(2)^.

### Construction of drug-disease and drug-target network

#### Cross-network construction

To construct the networks, we consider two different datasets. For the first one, the initial drug-disease interactions in domain $D_{1}$ are obtained from the Comparative Toxicogenomics Database (CTD): http://ctdbase.org/. For the second dataset, drug-disease relationships are obtained from Gottlieb et al. [33], which has been frequently used in many previous studies [12, 34] and is regraded as a gold standard dataset.

The CTD dataset contains 1, 048, 547 drug-disease associations. We only focus on those diseases that have OMIM https://www.omim.org/ identifiers for conveniently computing disease similarity scores later. We thus collect total of 1, 321 drugs, 3, 966 disease as well as 111, 481 drug-disease interactions.

The initial drug-target associations in domain $D_{2}$ can be directly obtained from DrugBank https://www.drugbank.ca/. We mainly focus on the approved small molecule compounds and require each drug to have at least two targets, resulting in 946 drugs, 3, 610 targets, and 10, 234 drug-target interactions. The requirement to have at least two targets for each drug is motivated by the notion that a drug can be used to treat a different disease most likely due to its off target activities. Across the two domains $D_{1}$ and $D_{2}$, we have 469 common drugs in the two networks. The statistics of these two networks are shown in Table 1.

**Table 1:** Statistics of drug-disease network (Domain 1) from CTD database and drug-target network (Domain 2) from DrugBank database. 469 drugs are overlapped between two networks in total.

Domain	|Drug|	|Target|	|Disease|	|Interaction|
Domain 1: drug-disease	1,321	-	3,966	111,481
Domain 2: drug-target	946	3,610	-	10,234

Dataset two is much smaller and only contains 1, 933 known drug-disease associations involving 593 drugs and 313 diseases. The drug-disease relationships in this dataset are human curated and are believed to be more reliable than the ones in the first dataset. We further collect the targets of those 593 drugs from the DrugBank database and construct the drug-target network that consists of 1, 011 targets and 3, 427 known drug-target interactions.

In addition to the cross links of the two heterogeneous networks, we also construct homogeneous edges and their weights based on the following similarity measures.

#### Drug-drug similarity

Although there are a number of measurements developed for computing drug-drug similarities, a recent study showed that Tanimoto coefficient similarity is highly efficient for fingerprint-based similarity measurement [35]. Chemical structures of drugs in Canonical SMILES form are directly downloaded from DrugBank. The Chemical Development Kit is then applied to compute the Tanimoto similarity score of any two drugs using their corresponding 2D chemical fingerprints [36]. Briefly, two drugs have a higher similarity score if they have more similar of chemical structures.

#### Target-target similarity

Protein targets consist of long chains of amino acid sequences, which perform a vast array of functions within organisms. Target-target similarity scores are thus calculated using the Smith-Waterman algorithm (e.g., 11 for gap open penaltyand 1 for its extension, BLOSUM62 for the scoring matrix.) based on their amino acid sequences. The similarity scores are then normalized into [0, 1] using the same method proposed in a previous work [16].

#### Disease-disease similarity

A recent study shows that a disease similarity defined based on the semantic similarity between MeSH terms describing the diseases is an accurate measure for heritable diseases at the molecular level [37]. The measure is defined based on the concept of information content of a MeSH term in an ontology, defined as the negative logarithm of the probability. Therefore, the disease similarity measure is unbounded, non-negative real number. We directly download their similarities from the Disimweb server http://www.paccanarolab.org/disimweb.

**Fig 2:**
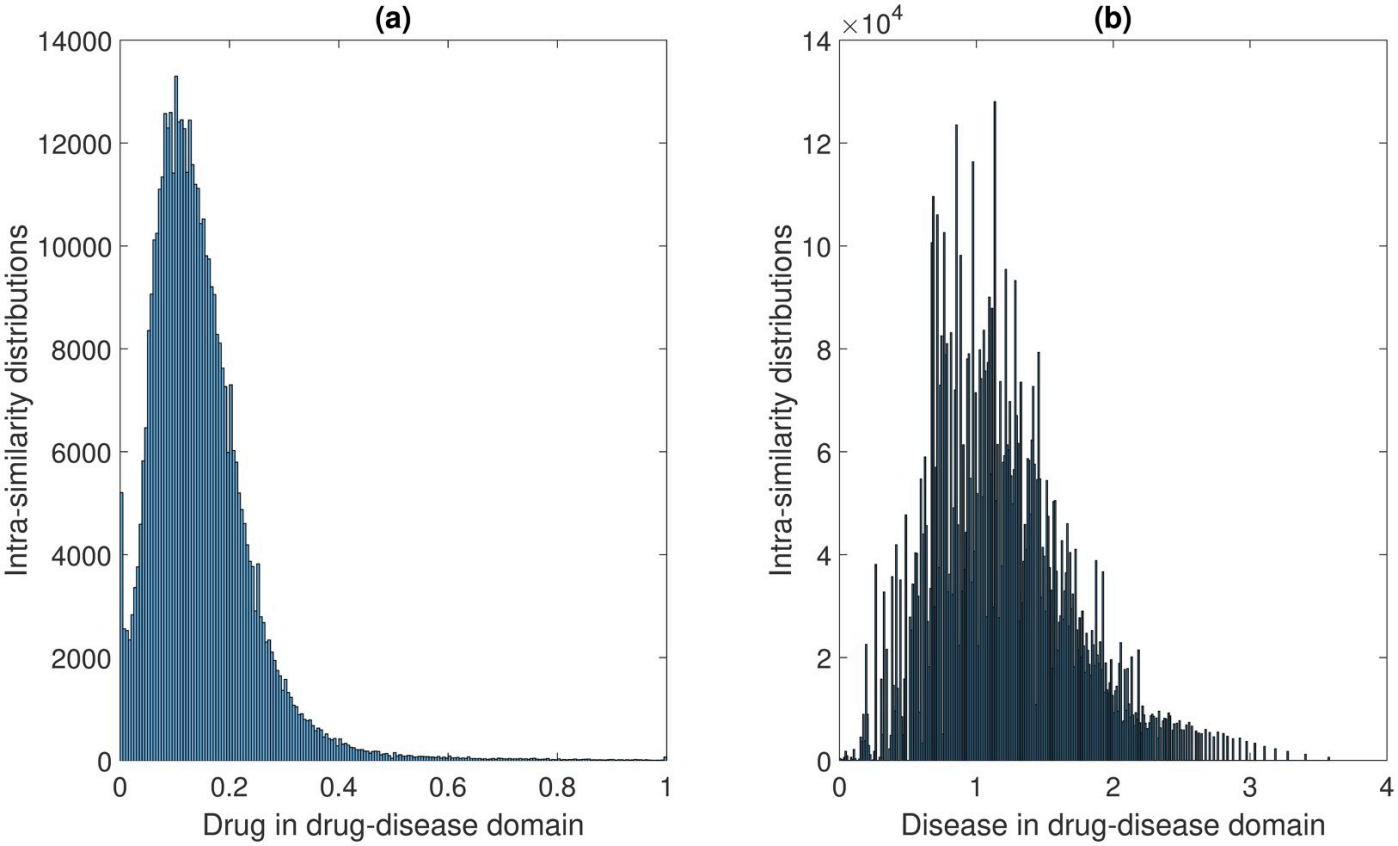
The intra-similarity distributions in drug-disease domain. (a) The intra-similarity distributions of drug pairs, the drug-drug similarities are calculated based on Tanimoto Score. (b) The intra-similarity distributions of disease pairs, the disease-disease similarities are computed based on the semantic similarity of MeSH terms. Note that all the self-similarity values of drugs and diseases have already been excluded in the histograms.

**Fig 3:**
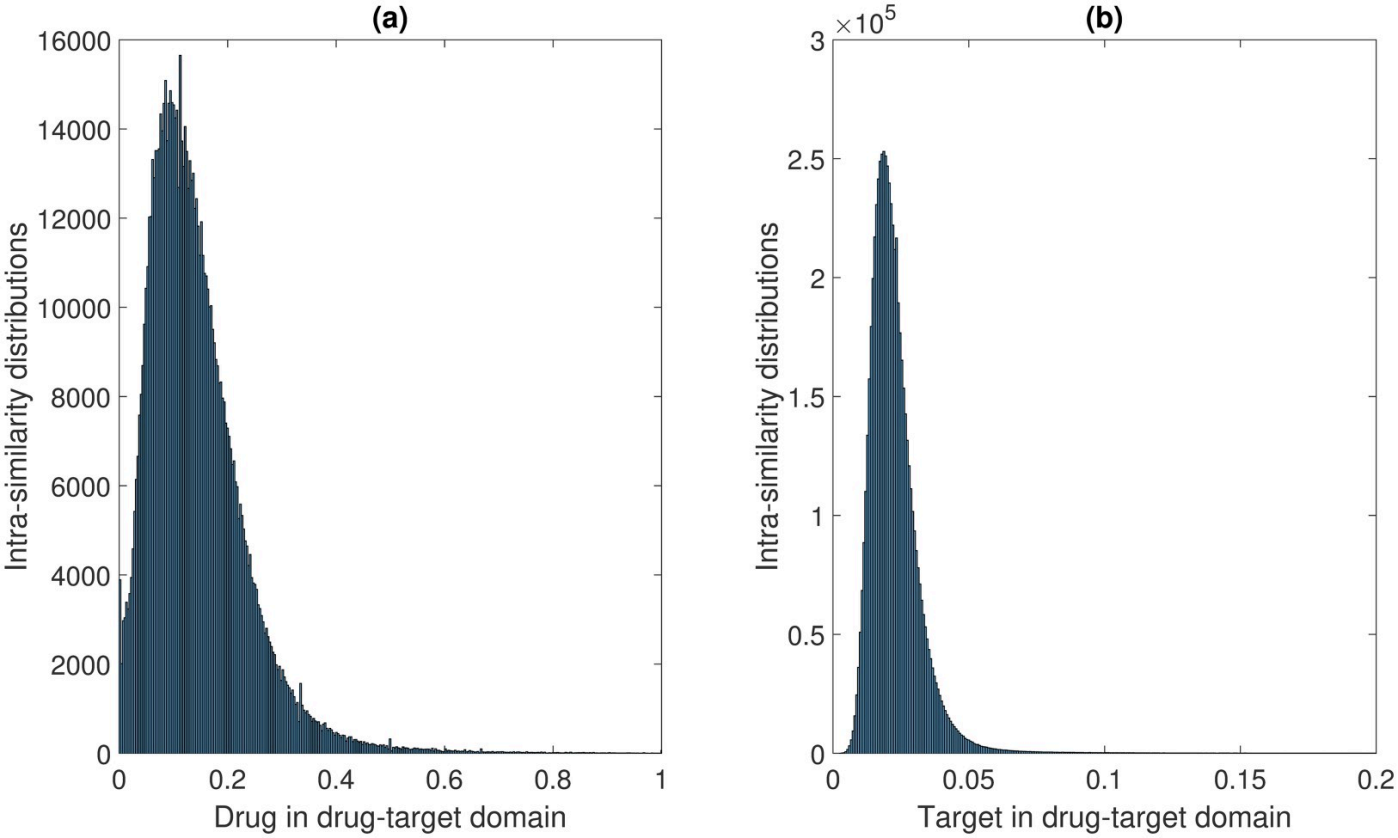
The intra-similarity distributions in drug-target domain. (a) The intra-similarity distributions of drug pairs, the drug-drug similarities are calculated based on Tanimoto Score. (b) The intra-similarity distributions of target pairs, the target-target similarities are calculated using the Smith-Waterman algorithm on target sequences. Note that all the self-similarity values of drugs and targets have already been excluded in the histograms. For target-target similarities, we only show the similarity values within [0, 0.2] since most of them are located in this range.

For the CTD dataset, the intra-similarity distributions of drug-drug and disease-disease are shown in Fig 2. The intra-similarity distributions of drug-drug and target-target are shown in Fig 3. The drug-drug similarity distributions from the two domains follow similar patterns. Overall, most drug pairs have similarities smaller than 0.4, which is not surprising given that most drugs may not be related. The disease similarities are not normalized. For two diseases, the lower their shared MeSH terms on the ontology, the higher the information content and their similarities. The target-target similarities are generally very small because of the diverse set of targets.

### The iDrug model

The key idea behind iDrug is to treat the problem as a cross-network embedding problem [38] by considering both within-network and cross-network relationships. We next provide more details for within-network factorization and cross-network consistency, and propose the unified model.

**1) Within-network factorization**: For a single-domain such as drug-disease prediction, we adopt the basic idea of graph regularized non-negative matrix factorization [38–40], which decomposes the drug-disease interaction matrix $X^{\left(1\right)}\in R^{n_{1}\times m_{1}}$ into two *r*_1_-rank feature matrices $U^{\left(1\right)}\in R^{n_{1}\times r_{1}}$ (i.e., drugs’ feature space) and $V^{\left(1\right)}\in R^{m_{1}\times r_{1}}$ (i.e., diseases’ feature space) by minimizing the following objective function:

$$\begin{array}{c} \begin{array}{cc} \underset{U^{\left(1\right)}\geq 0,V^{\left(1\right)}\geq 0}{\text{min}} & ∥W^{\left(1\right)}⊙\left(X^{\left(1\right)}-U^{\left(1\right)}{V^{\left(1\right)}}^{T}\right){∥}_{F}^{2}+\alpha \cdot \left(\text{Tr}\left({U^{\left(1\right)}}^{T}L_{u}^{\left(1\right)}U^{\left(1\right)}\right)+\text{Tr}\left({V^{\left(1\right)}}^{T}L_{v}^{\left(1\right)}V^{\left(1\right)}\right)\right) \end{array} \end{array}$$ (1)

where $L_{u}^{\left(1\right)}=D_{u}^{\left(1\right)}-A_{u}^{\left(1\right)}$, $L_{v}^{\left(1\right)}=D_{v}^{\left(1\right)}-A_{v}^{\left(1\right)}$ and ⊙ is the Hadamard product, Tr(⋅) is the Trace operator, ∥⋅∥_*F*_ is the matrix Frobenius norm and *α* is the regularization parameter. $D_{u}^{\left(1\right)}$ and $D_{v}^{\left(1\right)}$ are the diagonal degree matrices of $A_{u}^{\left(1\right)}$ and $A_{v}^{\left(1\right)}$, such as $D_{u}^{\left(1\right)}\left(i,i\right)=\sum_{j=1}^{n_{1}}A_{u}^{\left(1\right)}\left(i,j\right)$; $W^{\left(1\right)}\in R^{n_{1}\times m_{1}}$ is weight matrix indicating the weight of entities on **X**^(1)^. Typically, a smaller weight is assigned to unobserved samples. Based on the strategy for one-class collaborative filtering [38, 40], we set **W**^(1)^(*i*, *j*) = 1 if **X**^(1)^(*i*, *j*) is observed and **W**^(1)^(*i*, *j*) = *w* ∈ [0, 1) otherwise. We use trace optimization (i.e., the second term) to achieve within-network smoothness, which ensures that the low-rank representations of nodes *i* and *j* in the same layer will be close to each other. Finally, the non-negativity constraints on the factor matrices **U**^(1)^ and **V**^(1)^ lead to more interpretable results [41]. Similarly, for drug-target domain, we can decompose matrix $X^{\left(2\right)}\in R^{n_{2}\times m_{2}}$ into two *r*_2_-rank feature matrices $U^{\left(2\right)}\in R^{n_{2}\times r_{2}}$ (drugs’ feature space) and $V^{\left(2\right)}\in R^{m_{2}\times r_{2}}$ (targets’ feature space) in a similar way as the Eq (1).

**2) Cross-network consistency**: iDrug further captures cross-network relationships by making the hypotheses that the partial overlap of drugs are consistent with each other across the two domains because they all represent the same drugs. To achieve this goal, we introduce a drug mapping matrix $S^{\left(1,2\right)}\in R^{n_{2}\times n_{1}}$ to represent the anchor links cross domain $D_{1}$ and $D_{2}$. To be specific, **S**^(1,2)^(*i*, *j*) = 1 if the *i*-th row of **U**^(2)^ and *j*-th row of **U**^(1)^ represent the same drug; **S**^(1,2)^(*i*, *j*) = 0 otherwise. Note that at most one element in each row of **S**^(1,2)^ can be 1 because of the one-to-one constraint of anchor links across the two domains. We then observe that **S**^(1,2)^ **U**^(1)^ in fact project the partial overlap of drug feature space from domain $D_{1}$ to domain $D_{2}$. In addition, if two drugs are similar in domain $D_{1}$, they should also be similar after projecting to domain $D_{2}$. The cross-network consistency of the partial overlap of drugs can thus be achieved by minimizing the following disagreement [42]:

$$\begin{array}{c} D\left(U^{\left(1\right)},U^{\left(2\right)}\right)={∥S^{\left(1,2\right)}U^{\left(1\right)}{\left(S^{\left(1,2\right)}U^{\left(1\right)}\right)}^{T}-U^{\left(2\right)}{U^{\left(2\right)}}^{T}∥}_{F}^{2} \end{array}$$ (2)

In other words, the feature space **U**^(1)^ in the drug domain $D_{1}$ should match the feature space **U**^(2)^ in the drug domain $D_{2}$ as much as possible because of their overlapped drugs.

**3) The unified model**: Finally, we can integrate the domain-specific within-network objective with respect to drug-target and drug-disease in Eq (1), with the cross-network consistency Eq (2) into a unified objective function as follows:

$$\begin{array}{c} \begin{array}{cc} \text{min} & \;J=\underset{\text{domain}\;\text{factorization}}{\underset{︸}{\sum_{i=1}^{2}{∥W^{\left(i\right)}⊙\left(X^{\left(i\right)}-U^{\left(i\right)}{V^{\left(i\right)}}^{T}\right)∥}_{F}^{2}}}+\beta \underset{\;\text{cross-network}\;\text{consistency}}{\underset{︸}{∥S^{\left(1,2\right)}U^{\left(1\right)}{\left(S^{\left(1,2\right)}U^{\left(1\right)}\right)}^{T}-U^{\left(2\right)}{U^{\left(2\right)}}^{T}{∥}_{F}^{2}}}\\ + & \underset{\;\text{within-network}\;\text{smoothness}}{\underset{︸}{\alpha \sum_{i=1}^{2}\left(\text{Tr}\left({U^{\left(i\right)}}^{T}\left(D_{u}^{\left(i\right)}-A_{u}^{\left(i\right)}\right)U^{\left(i\right)}\right)+\text{Tr}\left({V^{\left(i\right)}}^{T}\left(D_{v}^{\left(i\right)}-A_{v}^{\left(i\right)}\right)V^{\left(i\right)}\right)\right)}}+\underset{\;\text{regularization}}{\underset{︸}{\gamma \sum_{i=1}^{2}(∥U^{\left(i\right)}{∥}_{1}+∥V^{\left(i\right)}{∥}_{1})}}\\ & \text{s}.\text{t}.\;U^{\left(i\right)}\geq 0,V^{\left(i\right)}\geq 0,\;\text{for}\;i=1,2 \end{array} \end{array}$$ (3)

where the *l*_1_-norm penalty on **U**^(*i*)^ and **V**^(*i*)^ (e.g., ∥**A**∥_1_ = ∑_*ij*_ **A**_*ij*_) can achieve a more sparse solution [43]. The regularization parameters *α*, *β*, and *γ* can adjust the relative importance of within-network smoothness, cross-network consistency, and sparseness of optimal solutions, which will be studied later. For convenience, all symbols in Eq (3) are summarized in Table 2.

**Table 2:** The symbols used in the objective function Eq (3) and their descriptions.

Symbol	Definition and Description
**X**^(1)^,**W**^(1)^	The adjacency matrix and the weight matrix of the known drug-disease interactions
**X**^(2)^,**W**^(2)^	The adjacency matrix and the weight matrix of the known drug-target interactions.
**U**^(1)^,**V**^(1)^	The low-rank representations of drugs and diseases in the drug-disease domain.
**U**^(2)^,**V**^(2)^	The low-rank representations of drugs and targets in the drug-target domain.
**S**^(1,2)^	The drug mapping matrix to denote anchor links across the two domains.
$A_{u}^{\left(1\right)},D_{u}^{\left(1\right)}$	The drug-drug similarity matrix and its degree matrix in the drug-disease domain.
$A_{u}^{\left(2\right)},D_{u}^{\left(2\right)}$	The drug-drug similarity matrix and its degree matrix in the drug-target domain.
$A_{v}^{\left(1\right)},D_{v}^{\left(1\right)}$	The target-target similarity matrix and its degree matrix in the drug-disease domain.
$A_{v}^{\left(2\right)},D_{v}^{\left(2\right)}$	The disease-disease similarity matrix and its degree matrix in the drug-target domain.
*n*_1_,*m*_1_	The number of drugs and diseases in the drug-disease domain.
*n*_2_,*m*_2_	The number of drugs and targets in the drug-target domain.
*r*_1_,*r*_2_	The ranks of matrices {**U**^(1)^, **V**^(1)^} and {**U**^(2)^, **V**^(2)^}.

### Learning algorithm

In this section, we provide rigorous theoretical analysis of iDrug in terms of its correctness, convergence, and complexity.

The objective function in Eq (3) is non-convex when considering all variables. We can optimize it by using the multiplicative update minimization approach [41], i.e., the objective function is alternately minimized with respect to one variable while fixing others. This procedure repeats until convergence, i.e., $∥J^{\left(t+1\right)}-J^{\left(t\right)}∥\leq \delta$, where *δ* is a small constant. The optimization procedure is summarized in S1 Fig. The details of the correctness and convergence of our algorithm can be found in the supplementary materials (S1 Appendix). Here we only include the updating formula of each variable as follows:

$$\begin{array}{c} \begin{array}{c} U^{\left(1\right)}\leftarrow U^{\left(1\right)}⊙\sqrt{\frac{X^{\left(1\right)}V^{\left(1\right)}+\alpha A_{u}^{\left(1\right)}U^{\left(1\right)}+2\beta \Delta }{T^{\left(1\right)}+\alpha D_{u}^{\left(1\right)}U^{\left(1\right)}+2\beta \Theta +0.5\gamma }} \end{array} \end{array}$$ (4)

$$\begin{array}{c} \begin{array}{c} U^{\left(2\right)}\leftarrow U^{\left(2\right)}⊙\sqrt{\frac{X^{\left(2\right)}V^{\left(2\right)}+\alpha A_{u}^{\left(2\right)}U^{\left(2\right)}+2\beta \Xi }{T^{\left(2\right)}+\alpha D_{u}^{\left(2\right)}U^{\left(2\right)}+2\beta U^{\left(2\right)}{U^{\left(2\right)}}^{T}U^{\left(2\right)}+0.5\gamma }} \end{array} \end{array}$$ (5)

$$\begin{array}{c} \begin{array}{c} V^{\left(i\right)}\leftarrow V^{\left(i\right)}⊙\sqrt{\frac{{X^{\left(i\right)}}^{T}U^{\left(i\right)}+\alpha A_{v}^{\left(i\right)}V^{\left(i\right)}}{(\left({R^{\left(i\right)}}^{T}+w^{2}V^{\left(i\right)}{U^{\left(i\right)}}^{T}\right)U^{\left(i\right)}+\alpha D_{v}^{\left(i\right)}V^{\left(i\right)}+0.5\gamma }} \end{array} \end{array}$$ (6)

where

$$\begin{array}{c} \Delta ={S^{\left(1,2\right)}}^{T}U^{\left(2\right)}{U^{\left(2\right)}}^{T}S^{\left(1,2\right)}U^{\left(1\right)}\\ \Theta ={S^{\left(1,2\right)}}^{T}S^{\left(1,2\right)}U^{\left(1\right)}{U^{\left(1\right)}}^{T}{S^{\left(1,2\right)}}^{T}S^{\left(1,2\right)}U^{\left(1\right)}\\ \Xi =S^{\left(1,2\right)}U^{\left(1\right)}{U^{\left(1\right)}}^{T}{S^{\left(1,2\right)}}^{T}U^{\left(2\right)}\\ R^{\left(i\right)}=\left(1-w^{2}\right)I^{\left(i\right)}⊙\left(U^{\left(i\right)}{V^{\left(i\right)}}^{T}\right)\\ T^{\left(i\right)}=\left(R^{\left(i\right)}+w^{2}U^{\left(i\right)}{V^{\left(i\right)}}^{T}\right)V^{\left(i\right)} \end{array}$$

and **I**^(*i*)^ is an indicator matrix for the observed elements in **X**^(*i*)^, i.e., **I**^(*i*)^(*u*, *v*) = 1 if **X**^(*i*)^(*u*, *v*) > 0, and **I**^(*i*)^(*u*, *v*) = 0 otherwise.

Once we solve Eq (3), for a give drug *i* and disease *j* in the drug repositioning domain, we can infer their potential association by ${\tilde{X}}^{\left(1\right)}\left(i,j\right)=U^{\left(1\right)}\left(i,:\right){V^{\left(1\right)}\left(j,:\right)}^{T}$. Similarly, for a give drug *i* and target *j* in the drug-target domain, the novel drug-target associations can be inferred by computing ${\tilde{X}}^{\left(2\right)}\left(i,j\right)=U^{\left(2\right)}\left(i,:\right){V^{\left(2\right)}\left(j,:\right)}^{T}$.

#### Complexity

According to the updating rules (Eqs (4) to (6)), the time complexity of our optimization algorithm is $O(k\cdot \left(nmr+n^{2}r+sr\right)+n^{3}+nr^{2})$, where *k* is the number of iterations, *n* = max{*n*_1_, *n*_2_}, *m* = max{*m*_1_, *m*_2_}, *r* = max{*r*_1_, *r*_2_}, and *s* = max{nnz(**X**^(1)^), nnz(**X**^(2)^)}, where nnz(**X**) is the number of non-zero elements in **X**. In practice, *r* ≪ min{*m*, *n*} and *s* is usually very small due to the sparsity of networks. Moreover, the $O(n^{3})$ term is from the matrix multiplication ${\text{S}}^{{\left(1,2\right)}^{T}}\;{\text{S}}^{\left(1,2\right)}$ in Eq (4). Since **S**^(1,2)^ is the very sparse mapping matrix and is unchanged in each iteration, we can thus cache ${\text{S}}^{{\left(1,2\right)}^{T}}\;{\text{S}}^{\left(1,2\right)}$ in advance to reduce the time complexity. The overall complexity of our algorithm can be denoted as $O(k\cdot \left(nmr+n^{2}r\right))$ in total. Although iDrug performs cross-domain learning using two biological networks, the computational complexity remains the same as the state-of-the-art matrix factorization algorithms in a single domain, such as GRMF [34] for drug-target prediction.

## Results

In this section, we conduct several experiments to evaluate the performance of our proposed iDrug on the two domains, respectively. Specifically, we perform the drug repositioning by fully using the knowledge containing in drug-target domain and vice versa in the cross-validation experiments. Many approaches exist for both problems. However, for some approaches, it is extremely challenging to compare their performance because these approaches are closely tied to the data that they are using. For example, the model structures are usually determined by the available data they have [15, 33]. In this study, we only compare four network-based methods that can easily take drug/target/disease similarities as inputs. The baseline methods are as follows:

- RLS-Kron [44], a kernel-based classifier that combines chemical and genomic similarity matrices for drug-target prediction.
- TL_HGBI [25]: a random-walk based algorithm for a three layers drug-target-disease network to predict the new interactions between drugs and diseases (targets).
- MBiRW [12]: a bi-random walk algorithm on a bipartite network to identify potential indications by further adjusting the clustering of drugs and diseases.
- GRMF [34]: a matrix factorization method that uses graph regularization to learn low-rank representations for drugs and targets.

Although some of them are originally designed for drug repositioning (e.g., TL_HGBI and MBiRW), all of above methods can be directly applied to the two domains as discussed before. The parameters of these algorithms are first initialized as those in the original paper and then tuned for the optimal performance. For RLS-Kron, the regularization parameter *σ* = 1 and the kernel bandwidths *γ* = 1. For random-walk based algorithm TL_HGBI and MBiRW, we set their thresholds the same as the optimal settings in their original papers. For GRMF, we tune the regularization parameter by using grid search algorithm [34], and λ_*l*_ = 0.5, λ_*d*_ = λ_*t*_ = 10^−3^ are chosen for the best performance. For iDrug, we set rank *r*_1_ = 90 and *r*_2_ = 70, weight in Eq (6) *w* = 0.3 and regularization parameters *α* = *β* = *γ* = 0.01. The impact of regularization parameters are presented in the Sensitivity Analysis subsection.

### Cross-validation for drug-disease prediction

In order to provide a complete picture of the performance for each approach, we conduct five-fold cross-validation experiments under the following three scenarios for drug-disease prediction [19, 34, 45]:

1. ‘pair prediction’ scenario, which predicts unknown interactions between known drugs and diseases. All known drug-disease associations are split into five folds, in which four folds are used for training and one fold for testing.
2. ‘new drug’ scenario, which predicts diseases for new drugs. In this scenario, the drugs are divided into five disjoint subsets. The drug-disease associations of four folds of drugs are used for training and the rest pairs are used for testing.
3. ‘new disease’ scenario, which predicts drugs for new diseases. The setting is similar to scenario two, but data are separated based on diseases.

For scenario 1, the original drug-disease associations are very sparse with a large fraction of unknown interactions. We choose one fold of known pair interactions as positive samples and further randomly select an equal number of unknown interactions as negative samples as test set. The four folds of known interactions and the rest of unknown pairs are used to train the model [19, 21]. By varying the rank threshold, we can calculate various true positive rate (TPR), false positive rate (FPR), Precision and Recall values. We then use Area Under the Receiver Operating Characteristic curve (AUROC) and Area Under the Precision Recall curve (AUPR) to assess the performance of different models [19, 34, 45].

For scenarios 2 and 3, we evaluate different methods mainly based on the precision of top-*k* metric because we are more interested in the top-*k* ranked candidates for ‘new drug’ or ‘new disease’ in the drug discovery process [6, 7]. Note that the entire drug-target associations are all incorporated when performing the task of drug repositioning. For each method, five-fold cross-validation experiments are repeated 10 times independently and the average performance is reported.

#### Experimental results

**Fig 4:**
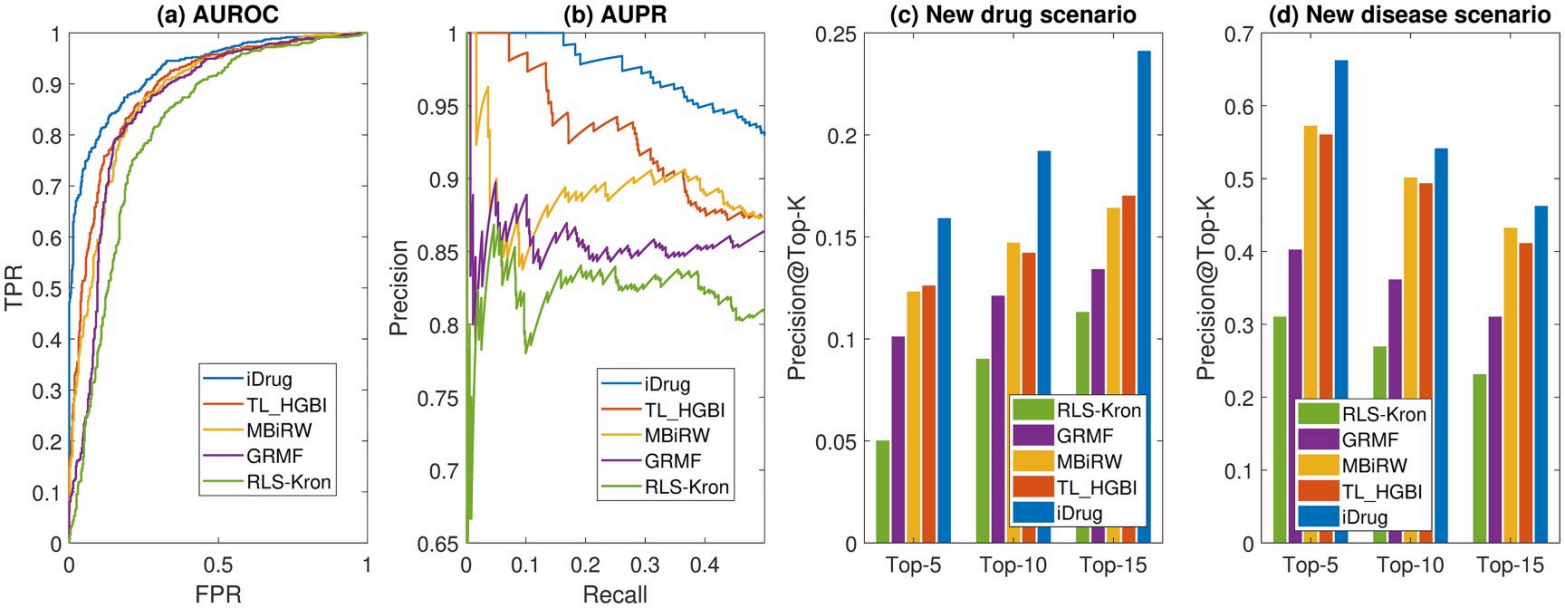
Comparison on the performance of different methods on drug repositioning. (a) The AUROC curves for the ‘pair prediction’ scenario. (b) The AUPR curves for the ‘pair prediction’ scenarios. (c) Precision of the top-*k* candidates for the ‘new drug’ scenario. (d) Precision of the top-*k* candidates for the ‘new disease’ scenario.

Our results (Fig 4) clearly show that the proposed iDrug consistently outperforms all the other approaches for all three scenarios. Fig 4(a) shows that iDrug achieves an AUROC value of 0.9213, which is better than that of the other methods in the same experimental scenario (e.g., TL_HGBI: 0.886, MBiRW: 0.879, GRMF: 0.863, and RLS-Kron: 0.844). Meanwhile, iDrug achieves an AUPR of 0.938, which are higher than all the other approaches (TL_HGBI: 0.881, MBiRW: 0.876, GRMF: 0.847, and RLS-Kron: 0.813). Several interesting observations can be made based on the results in Fig 4. First, iDrug and TL_HGBI, both of which integrate target information, perform better than the rest methods, indicating the contributions of target information for drug-disease prediction. In a previous study [25], it was shown that the performance gradually decreased when some of the observed drug-target links in the network were removed randomly. Our results here further confirm that it is preferable to jointly model drug-target-disease relationships to better understand drug’s MoAs. Second, comparing iDrug with TL_HGBI, TL_HGBI requires a common set of drug nodes across the two domains, therefore can be viewed as a sub-network of iDrug and is more prone to the issue of data sparsity. For instance, for a new compound added to the network, its similarity with existing drugs can be calculated. However, its interactions with diseases and targets might be completely unknown. TL_HGBI cannot address such a *cold-start* problem for novel drug discovery. In contrast, iDrug overcomes this issue by jointly learning on larger drug-disease and drug-target networks, and transferring domain-specific knowledge through anchor links cross domains [27]. Larger networks tend to contain more information thus ease the issue of data sparsity. Therefore, iDrug is expected to perform better as shown here. In comparing iDrug with MBiRW, both approaches employ the concept of drug community/cluster to improve their performance, but in different ways. The community of drugs in MBiRW was constructed based on common drug indications, which highly depended on the known drug-disease associations and might suffer from bias from data. Different from MBiRW, iDrug restricts the community of drugs by imposing consistency constraints among drugs from the two domains, i.e., *D*(**U**^(1)^, **U**^(2)^). It can therefore obtain more reliable community of drugs for both domains. iDrug achieves higher AUPR score than GRMF, presumably due to the fact that GRMF only incorporates information of drugs and diseases. In fact, GRMF can be viewed as a degraded version of iDrug for single-domain prediction since they both try to learn the low-rank representations of drugs and diseases via matrix factorization with graph regularization. iDrug improves the accuracy by simply transferring knowledge from multiple domains. Finally, the kernel-based RLS-Kron model performs the worst among all the approaches since it is often hard to choose an appropriate kernel function, which usually requires more specific domain knowledge from the experts.

For scenarios 2 and 3 (Fig 4(c) and 4(d)), iDrug also perform better than the other approaches in term of the precision of top-5, top-10 and top-15 metrics. It is interesting to notice the difference in the two scenarios. The precision of top-*k* candidates In the ‘new drug’ scenario is much lower than the precision in the ‘new disease’ scenario. We suspect that one of the main reasons is that the number of overlapped drugs connecting the two domains is decreased when splitting the drugs into five disjoint sets in the ‘new drug’ scenario. The impact of anchor links is thus reduced and weak domain-specific knowledge can be transferred cross domains in the cross-validation experiments. In the ‘new disease’ scenario, the anchor links are preserved and rich domain-specific knowledge are transferred cross domains, resulting in a higher precision.

### Cross-validation for drug-target prediction

In this section, we test and compare iDrug’s performance with other approaches for the task of drug-target prediction. Similar to drug-disease prediction, we conduct five-fold cross-validation experiments under three scenarios: ‘pair prediction’, ‘new drug’, and ‘new target’ scenarios. The entire drug-disease network is preserved during the learning process. The experiments are also repeated 10 times independently and the average scores are reported. For the task of drug-target prediction, the performance is obtained by setting *α* = *β* = 0.01 and *γ* = 0.001.

**Fig 5:**
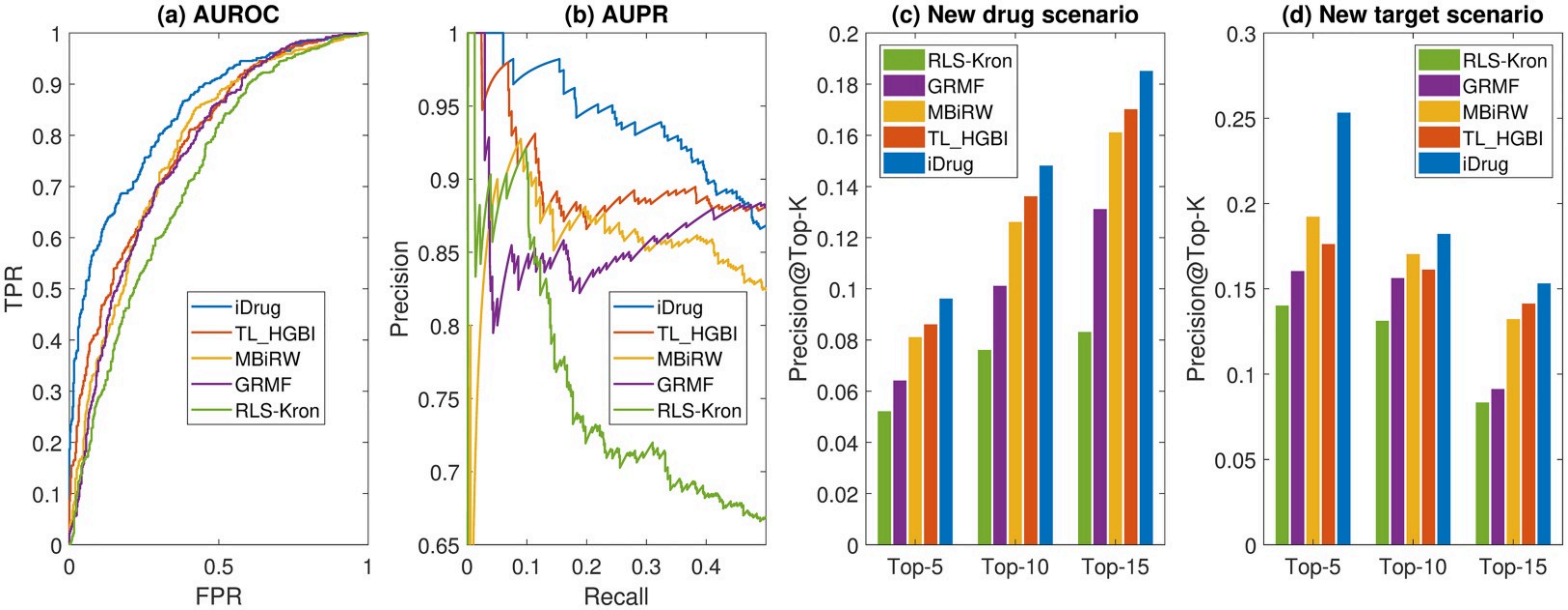
Performance comparison of different methods on drug-target prediction. (a) The AUROC curves for the ‘pair prediction’ scenario. (b) The AUPR curves for the ‘pair prediction’ scenario. (c) Precision of the top-*k* candidates for the ‘new drug’ scenario. (d) Precision of the top-*k* candidates for the ‘new target’ scenario.

Results show that iDrug consistently outperforms all other methods in all three scenarios for drug-target prediction for all the measures (Fig 5). For instance, iDrug achieves a 0.897 AUPR score, much higher than that of the other approaches. The next two closest competitors are TH_HGBI (AUPR: 0.856) and MBiRW (AUPR: 0.849). In terms of the precision of top-*k* metric, iDrug is also able to better predict candidates for novel drugs and novel targets, for the same reason as we discussed in drug-disease prediction experiments. The superior performance of iDrug demonstrates its potential on transferring knowledge across two related domains, thus serving as a promising tool for drug-target prediction.

### Sensitivity analysis

There are six hyper parameters in our proposed framework: *r*_1_, *r*_2_, *α*, *β*, *γ* and *w*. For the ranks of latent matrices (*r*_1_ and *r*_2_), intuitively, the greater the ranks are, the more latent information can be captured, while the higher the computational costs are. In the experiments, we find that *r*_*i*_ ≤ 0.1 min{*n*_*i*_, *m*_*i*_} can achieve a good trade-off between accuracy and running time. We thus set *r*_1_ = 90 and *r*_2_ = 70 in the sensitivity analysis of other parameters. Here we present the results of the impacts of these parameters for the task of drug repositioning using the AUPR measurement in scenario 1. The impacts of the regularization parameters for different scenarios (e.g., using precision of top-*k* candidates) in different domain (e.g., drug-target prediction) can be conducted in a similar fashion. We omit the details here.

#### The impact of *α*, *β*, and *γ*

As discussed earlier, *α*, *β*, and *γ* represent the contributions of within-domain smoothness, cross-network consistency, and the sparseness of solutions, respectively. We fix one of three parameters, and study the impacts of the remaining two on the inference results while fixing all the other parameters (*w* = 0.3, *r*_1_ = 90 and *r*_2_ = 70). For example, we first fix *γ* = 0.01 and vary *α* and *β* within {10^−4^, 10^−3^, 10^−2^, 10^−1^, 10^0^} by using the grid-search technique. Results in S2 Fig show that AUPR is very stable across the wide range for both *α* and *β* in the task of drug-disease prediction: all AUPR scores are over 0.910. Similar patterns are observed in studying the impacts of (*α*, *γ*) and (*β*, *γ*) pairs (S3 and S4 Figs, respectively). In general, a relatively high AUPR score can be achieved when *α* = *β* = *γ* ≈ 0.01.

#### The impact of *w*

The parameter *w* ∈ (0, 1) in the weight matrix **W** denotes the cost assigned to unobserved samples, which is very useful for imbalance datasets [38, 40]. To perform sensitivity analysis, we fix *r*_1_ = 90, *r*_2_ = 70 and *α* = *β* = *γ* = 0.01, and vary *w* within {0.1, 0.2, 0.3, …, 0.9}. The AUPR measure for drug-disease prediction is used to evaluate the performance. S5 Fig shows that iDrug is very robust to the regularization parameter *w* within (0, 1).

Finally, S6 Fig shows the rate of convergence of our optimization algorithm in the experiments. The values of the objective function in Eq (3) steadily decrease with more iterations and less than 100 iterations are sufficient for convergence.

### Experimental results on a gold standard dataset

We further investigate the performance of various methods on a human curated dataset initially studied by Gottlieb et al. [33], which has been commonly used in many previous studies [12, 34]. This dataset only contains 1, 933 known drug-disease associations involving 593 drugs and 313 diseases. We further collect all the 1, 011 targets of those 593 drugs from DrugBank database, which consists of 3, 427 drug-target interactions. We evaluate different methods for the ‘pair prediction’ scenario for both drug-disease and drug-target predictions.

**Fig 6:**
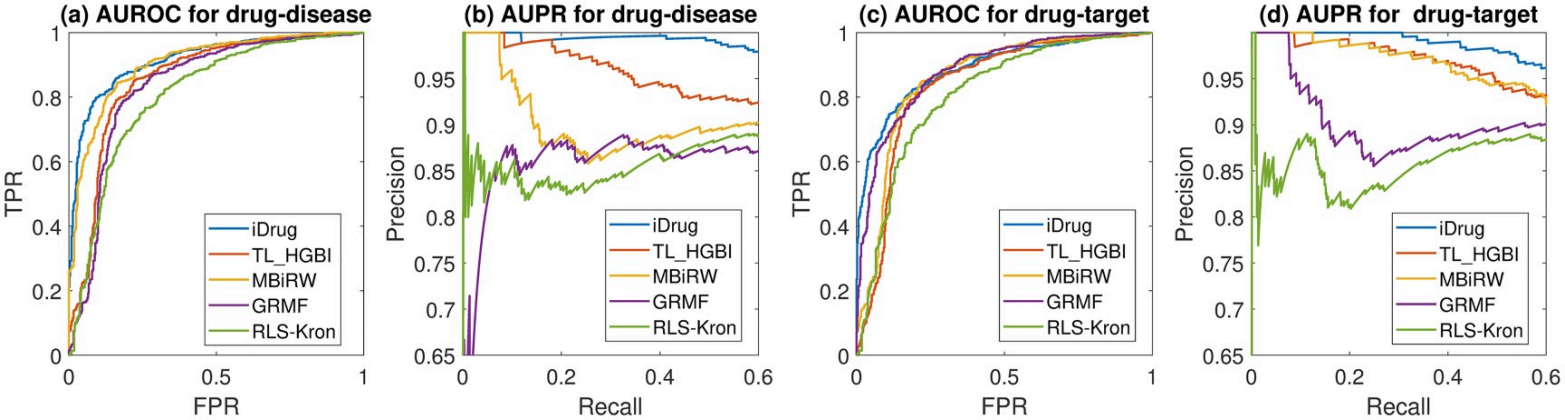
Performance comparison of different methods for the ‘pair prediction’ scenario on the gold standard dataset. (a) The AUROC curves for drug-disease prediction. (b) The AUPR curves for drug-disease prediction. (c) The AUROC curves for drug-target prediction. (d) The AUPR curves for drug-target prediction.

Results in Fig 6 show that iDrug performs better than the rest of the algorithms for both tasks using the gold standard dataset. For example, for drug-disease prediction, iDrug achieves 0.917 for AUROC and 0.926 for AUPR, which are higher than the two measures from any other methods and are consistent with the results based on the CTD dataset. It is also observed that comparing with other approaches, iDrug achieves the greatest improvement in terms of AUPR. The next two closest competitors are TH_HGBI (AUPR: 0.883) and MBiRW (AUPR:0.846). An explanation is that AUPR punishes highly ranked false positives much more than AUROC does, especially for sparse dataset [46]. Similar trends can be observed for the task of drug-target prediction.

Notice that the dataset from Gottlieb et al. [33] was collected almost a decade ago. All the drugs, diseases, and drug-disease interactions in the dataset can be found in the CTD dataset. Therefore, we can actually compare our novel prediction results based on the small dataset with the ones actually exist in the CTD dataset but not in the small dataset, which can be viewed as an indication of how iDrug might work in reality in predicting new drug-disease relationships. Towards that end, we collect the top 20 drugs for all the 313 diseases, and identify 514 new drug-disease interactions that can be found in the CTD dataset. Based on the hypergeometric distribution (all possible drug-disease pairs in CTD is 5239086, the number of positive pairs in CTD is 111481, the total number of drug-disease pairs considered 6260 and the number of positive pairs is 514), the result is extremely significant (*p*-value is 6.19 × 10^−144^) and shows that the top ranked predictions by iDrug are greatly enriched in the CTD dataset.

## Discussion

Drug repositioning and drug-target prediction have been widely recognized as promising tasks to better understand drug’s MoAs. Existing machine learning methods often consider them as two isolated tasks and ignore the potential dependencies between these two related domains. In this paper, we propose a new learning framework, iDrug, which seamlessly integrates drug repositioning and drug-target prediction into a unified model. iDrug treats the problem as a cross-network embedding problem by considering both of within-network and cross-network relationships. We also develop the optimization algorithm to solve the problem and provide rigorous theoretical analysis concerning its correctness, convergence and complexity. Experimental results on both tasks of drug repositioning and drug-target prediction demonstrate that the proposed iDrug outperforms existing algorithms in all cases for both drug-disease and drug-target prediction. The efficiency and effectiveness of iDrug allows us to better understand new biomedical insights of drug-target-disease in drug discovery.

Our approach can be further improved in several directions. For example, although our model considers rich bioinformatics and cheminformatics data from publicly available databases, data quality can not be guaranteed and the network data may be incomplete and contain noise. Even with existing data, data representations, for example using binary fingerprints to represent drug chemical structures, can have significant impact on the prediction performance [33]. To alleviate the problem, one direction of future work is to incorporate more heterogeneous data sources describing drugs, targets, and diseases so that multiple data sources may provide complementary information to allow missing data imputation and noise removal. Other heterogeneous data sources, such as drug’s side-effect, drug’s ligand binding site information, target’s Gene Ontology annotations, disease pathways, and diseases’ Human Phenotype Ontology, can also be integrated into the heterogeneous network for the two tasks [14, 18, 19, 21, 24, 47]. One possible extension is to use coupled matrix factorization to jointly capture the low-rank representations of the network and multiple data sources simultaneously.

Additionally, iDrug is built upon the matrix factorization framework, which approximates unobserved values using linear combinations of latent features. It is therefore can not capture more complex and non-linear drug-disease or drug-target interactions in the latent space. To overcome the linearity of iDrug, we will investigate the application of deep learning techniques, which have shown some initial success in capturing more complex and non-linear feature interactions in medicine and biology [48, 49]. One possible strategy is to train an autoencoder in an unsupervised way to capture the nonlinear feature representations of drugs, targets, and diseases. These nonlinear features can be integrated to identify novel drug-disease and drug-target interactions by using deep cross-network embedding techniques. Another inherent limitation for all factorization based approaches is the interpretability. Some existing works use heterogeneous knowledge graphs that incorporate many different types of data and identify paths from the graphs to interpret identified relationships [24]. However, it is a challenge task how one can combine these two strategies into one unified framework and is worthy further investigations.

More recently, a growing number of studies have suggested that non-coding RNAs (ncRNAs), especially microRNAs (miRNAs), play a significant role in affecting gene expressions and in disease progressions, making them a new class of drug targets [47, 50, 51]. Therefore, it becomes important to understand the relationship between drugs and miRNA targets. Theoretically, the framework proposed here can be applied to miRNA targets by defining a proper miRNA-miRNA similarity. One challenge is that knowledge about existing drug-miRNA interactions is limited. It is an interesting question to explore how to adopt existing approaches including iDrug to identify novel drug-miRNA interactions [50, 52].

Finally, most existing work including iDrug, implicitly assuming the monotherapy strategy in investigating drug-target-disease relationships, cannot easily incorporate polypharmacology or polytherapy strategy. On the other hand, polypharmacology and polytherapy, offer many advantages compared to monotherapy [53–56], including better efficacy, lower individual dosage, and reduced adverse effects. As an example of potential polytherapy, pentamidine and chlorpromazine show no anti-tumor activities when being administrated individually, but their combination inhibits tumor growth more effectively than paclitaxel, an anticancer chemotherapy drug. Furthermore, drug combinations often use existing drugs that have been approved by the Food and Drug Administration (FDA). Therefore, their toxic properties and side effects are usually well studied, and their combination could be directly used safely by patients [53–56]. For future work, we aim to extend our drug layers to contain drug pairs to study drug synergy for complex diseases. Eventually, wet lab experimental testing is a necessary step to validate outcomes of any computational approaches, which cannot be done without collaborations with investigators with expertise in biochemistry and drug development.

## Supporting information

S1 Appendix Optimization algorithm. The details of the optimization algorithm for solving **U**^(*i*)^ and **V**^(*i*)^, as well as its correctness and convergence results can be found here.
(PDF)

S1 Fig The pseudocode of the proposed iDrug. The algorithm to solve the objective function in Eq (3).
(TIF)

S2 Fig The impact of *α* and *β*. Grid-based search method to study the impact of *α* and *β* with respect to the AUPR measurement for the task of drug repositioning, while *γ* is fixed to be 0.01.
(TIF)

S3 Fig The impact of *α* and *γ*. Grid-based search method to study the impact of *α* and *γ* with respect to the AUPR measurement for the task of drug repositioning, while *β* is fixed to be 0.01.
(TIF)

S4 Fig The impact of *β* and *γ*. Grid-based search method to study the impact of *β* and *γ* with respect to the AUPR measurement for the task of drug repositioning, while *α* is fixed to be 0.01.
(TIF)

S5 Fig The impact of *w*. The impact of *w* with respect to the AUPR measurement for the task of drug repositioning.
(TIF)

S6 Fig Convergence on empirical data. The convergence of iDrug on empirical data for the task of drug repositioning.
(TIF)
