# Supplementary material for: iDrug: Integration of drug repositioning and drug-target prediction via cross-network embedding
Source: PLoS Comput Biol. 2020 Jul 15;16(7):e1008040. doi: 10.1371/journal.pcbi.1008040 (PMC7384678; doi:10.1371/journal.pcbi.1008040)
Supplement: S1 Appendix — The details of the optimization algorithm for solving U(i) and V(i), as well as its correctness and convergence results can be found here. (PDF) [file pcbi.1008040.s001.pdf]

Supplementary material for integration of drug repositioning and drug-target prediction via cross-network embedding

Huiyuan Chen[1], Feixiong Cheng[2,3,4], Jing Li[1,4*]

1 Department of Computer and Data Sciences, Case Western Reserve University, Cleveland, OH, USA.
2 Genomic Medicine Institute, Lerner Research Institute, Cleveland Clinic, Cleveland, OH, USA.
3 Department of Molecular Medicine, Cleveland Clinic Lerner College of Medicine, Case Western Reserve University, Cleveland, OH, USA.
4 Case Comprehensive Cancer Center, Case Western Reserve University School of Medicine, Cleveland, Ohio, USA.

* jingli@cwru.edu

Optimization solution

We proof the detailed of solution for our objective function Eq. (3) in the main paper according to the Karush-Kuhn-Tucker (KKT) condition [1].

1). Update $\mathbf{U}^{(1)}$

We formulate the Lagrange function with respect to $\mathbf{U}^{(1)}$ as

$$\begin{aligned} L(\mathbf{U}^{(1)}) =& \|\mathbf{W}^{(1)} \odot (\mathbf{X}^{(1)} - \mathbf{U}^{(1)}{\mathbf{V}^{(1)}}^T)\|_F^2 + \alpha \operatorname{Tr}({\mathbf{U}^{(1)}}^T(\mathbf{D}_u^{(1)} - \mathbf{A}_u^{(1)})\mathbf{U}^{(1)}) \\ &+\beta\|\mathbf{S}^{(1,2)}\mathbf{U}^{(1)}(\mathbf{S}^{(1,2)}\mathbf{U}^{(1)})^T - \mathbf{U}^{(2)}{\mathbf{U}^{(2)}}^T\|_F^2 + \gamma\|\mathbf{U}^{(1)}\|_1 \end{aligned} \tag{1}$$

The partial derivative of Lagrange function with respect to $\mathbf{U}^{(1)}$

$$\begin{aligned} \nabla_{\mathbf{U}^{(1)}} L =& -2(\mathbf{W}^{(1)} \odot \mathbf{W}^{(1)} \odot \mathbf{X}^{(1)})\mathbf{V}^{(1)} + 2(\mathbf{W}^{(1)} \odot \mathbf{W}^{(1)} \odot (\mathbf{U}^{(1)}{\mathbf{V}^{(1)}}^T))\mathbf{V}^{(1)} \\ &+ 2\alpha\mathbf{D}_u^{(1)}\mathbf{U}^{(1)} - 2\alpha\mathbf{A}_u^{(1)}\mathbf{U}^{(1)} - 4\beta{\mathbf{S}^{(1,2)}}^T\mathbf{U}^{(2)}{\mathbf{U}^{(2)}}^T\mathbf{S}^{(1,2)}\mathbf{U}^{(1)} \\ &+ 4\beta{\mathbf{S}^{(1,2)}}^T\mathbf{S}^{(1,2)}\mathbf{U}^{(1)}{\mathbf{U}^{(1)}}^T{\mathbf{S}^{(1,2)}}^T\mathbf{S}^{(1,2)}\mathbf{U}^{(1)} + \gamma\mathbf{E}_{n_1\times r_1} \end{aligned}$$

where $\mathbf{E}_{n_1\times r_1}$ is the matrix whose elements are all one with the size $n_1 \times r_1$. Using the KKT complementarity condition for the non-negative constraint on $\mathbf{U}^{(1)}$, we have $\nabla_{\mathbf{U}^{(1)}} L \odot \mathbf{U}^{(1)} = 0$, which lead to leads to the updating rule for $\mathbf{U}^{(1)}$

$$\mathbf{U}^{(1)} \leftarrow \mathbf{U}^{(1)} \odot \sqrt{\frac{(\mathbf{W}^{(1)} \odot \mathbf{W}^{(1)} \odot \mathbf{X}^{(1)})\mathbf{V}^{(1)} + \alpha\mathbf{A}_u^{(1)}\mathbf{U}^{(1)} + 2\beta\Delta}{(\mathbf{W}^{(1)} \odot \mathbf{W}^{(1)} \odot (\mathbf{U}^{(1)}{\mathbf{V}^{(1)}}^T))\mathbf{V}^{(1)} + \alpha\mathbf{D}_u^{(1)}\mathbf{U}^{(1)} + 2\beta\mathbf{\Theta} + 0.5\gamma\mathbf{E}_{n_1\times r_1}}} \tag{2}$$

where $\Delta = {\mathbf{S}^{(1,2)}}^T\mathbf{U}^{(2)}{\mathbf{U}^{(2)}}^T\mathbf{S}^{(1,2)}\mathbf{U}^{(1)}$ and $\mathbf{\Theta} = {\mathbf{S}^{(1,2)}}^T\mathbf{S}^{(1,2)}\mathbf{U}^{(1)}{\mathbf{U}^{(1)}}^T{\mathbf{S}^{(1,2)}}^T\mathbf{S}^{(1,2)}\mathbf{U}^{(1)}$ The optimal solution can be speed-up by the follows. Recall that we set $\mathbf{W}^{(1)}(u,v) = 1$ when $\mathbf{X}^{(1)}(u,v) > 0$ and $\mathbf{W}^{(1)}(u,v) = w$ when $\mathbf{X}^{(1)}(u,v) = 0$. Here, we can further define $\mathbf{I}^{(1)}$ as an indication matrix for the

observed element in $\mathbf{X}^{(1)}$, which is $\mathbf{I}^{(1)}(u,v)=1$ if $\mathbf{X}^{(1)}(u,v)>0$, and $\mathbf{W}^{(1)}(u,v)=0$ when $\mathbf{X}^{(1)}(u,v)=0$. Then the sparse matrix whose entities are estimated over the observed data can be computed as $\mathbf{R}^{(1)}=(1-w^2)\mathbf{I}^{(1)}\odot(\mathbf{U}^{(1)}\mathbf{V}^{(1)^T})$. With these notations, we can simplify the update rule as follows:

$$\mathbf{U}^{(1)} \leftarrow \mathbf{U}^{(1)} \odot \sqrt{\frac{\mathbf{X}^{(1)}\mathbf{V}^{(1)} + \alpha\mathbf{A}_u^{(1)}\mathbf{U}^{(1)} + 2\beta\Delta}{\mathbf{T}^{(1)}\mathbf{V}^{(1)} + \alpha\mathbf{D}_u^{(1)}\mathbf{U}^{(1)} + 2\beta\mathbf{\Theta} + 0.5\gamma}} \tag{3}$$

where $\mathbf{T}^{(1)}=\mathbf{R}^{(1)}+w^2\mathbf{U}^{(1)}\mathbf{V}^{(1)^T}$; Note that $\frac{[\cdot]}{[\cdot]}$ is the element-wise operator and the notation of $\mathbf{E}_{n_1\times r_1}$ can sometimes be omitted due to the broadcasting of the matrix and scalar addition operator.

2). Update $\mathbf{U}^{(2)}$

Similarly, we can define the Lagrange function with $\mathbf{U}^{(2)}$ as

$$\begin{aligned} L(\mathbf{U}^{(2)}) = & \|\mathbf{W}^{(2)}\odot(\mathbf{X}^{(2)}-\mathbf{U}^{(2)}\mathbf{V}^{(2)^T})\|_F^2 + \alpha\,\mathrm{Tr}(\mathbf{U}^{(2)^T}(\mathbf{D}_u^{(2)}-\mathbf{A}_u^{(2)})\mathbf{U}^{(2)}) \\ & + \beta\|\mathbf{S}^{(1,2)}\mathbf{U}^{(1)}(\mathbf{S}^{(1,2)}\mathbf{U}^{(1)})^T - \mathbf{U}^{(2)}\mathbf{U}^{(2)^T}\|_F^2 + \gamma\|\mathbf{U}^{(2)}\|_1 \end{aligned}$$

The partial derivative of Lagrange function with respect to $\mathbf{U}^{(1)}$

$$\begin{aligned} \nabla_{\mathbf{U}^{(2)}}L = & -2(\mathbf{W}^{(2)}\odot\mathbf{W}^{(2)}\odot\mathbf{X}^{(2)})\mathbf{V}^{(2)} + 2(\mathbf{W}^{(2)}\odot\mathbf{W}^{(2)}\odot(\mathbf{U}^{(2)}\mathbf{V}^{(2)^T}))\mathbf{V}^{(2)} + 2\alpha\mathbf{D}_u^{(2)}\mathbf{U}^{(2)} \\ & -2\alpha\mathbf{A}_u^{(2)}\mathbf{U}^{(2)} - 4\beta\mathbf{S}^{(1,2)}\mathbf{U}^{(1)}\mathbf{U}^{(1)^T}\mathbf{S}^{(1,2)^T}\mathbf{U}^{(2)} + 4\beta\mathbf{U}^{(2)}\mathbf{U}^{(2)^T}\mathbf{U}^{(2)} + \gamma\mathbf{E}_{n_2\times r_2} \end{aligned}$$

The updating rule for $\mathbf{U}^{(2)}$ can be obtained by the KKT complementarity condition as

$$\mathbf{U}^{(2)} \leftarrow \mathbf{U}^{(2)} \odot \sqrt{\frac{\mathbf{X}^{(2)}\mathbf{V}^{(2)} + \alpha\mathbf{A}_u^{(2)}\mathbf{U}^{(2)} + 2\beta\mathbf{S}^{(1,2)}\mathbf{U}^{(1)}\mathbf{U}^{(1)^T}\mathbf{S}^{(1,2)^T}\mathbf{U}^{(2)}}{(\mathbf{R}^{(2)}+w^2\mathbf{U}^{(2)}\mathbf{V}^{(2)^T})\mathbf{V}^{(2)} + \alpha\mathbf{D}_u^{(2)}\mathbf{U}^{(2)} + 2\beta\mathbf{U}^{(2)}\mathbf{U}^{(2)^T}\mathbf{U}^{(2)} + 0.5\gamma}} \tag{4}$$

where $\mathbf{R}^{(2)}=(1-w^2)\mathbf{I}^{(2)}\odot(\mathbf{U}^{(2)}\mathbf{V}^{(2)^T})$.

3). Update $\mathbf{V}^{(i)}$

The Lagrange function with respect to $\mathbf{V}^{(i)}$ (for $i=1,2$) as

$$L(\mathbf{V}^{(i)}) = \|\mathbf{W}^{(i)}\odot(\mathbf{X}^{(i)}-\mathbf{U}^{(i)}\mathbf{V}^{(i)^T})\|_F^2 + \alpha\,\mathrm{Tr}(\mathbf{V}^{(i)^T}(\mathbf{D}_v^{(i)}-\mathbf{A}_v^{(i)})\mathbf{V}^{(i)}) + \gamma\|\mathbf{V}^{(i)}\|_1$$

The partial derivative of Lagrange function with respect to $\mathbf{V}^{(i)}$

$$\begin{aligned} \nabla_{\mathbf{V}^{(i)}}L = & -2(\mathbf{W}^{(i)^T}\odot\mathbf{W}^{(i)^T}\odot\mathbf{X}^{(i)^T})\mathbf{U}^{(i)} + 2(\mathbf{W}^{(i)^T}\odot\mathbf{W}^{(i)^T}\odot(\mathbf{V}^{(i)}\mathbf{U}^{(i)^T}))\mathbf{U}^{(i)} \\ & + 2\alpha\mathbf{D}_v^{(i)}\mathbf{V}^{(i)} - 2\alpha\mathbf{A}_v^{(i)}\mathbf{V}^{(i)} + \gamma\mathbf{E}_{m_i\times r_i} \end{aligned}$$

the updating rule for $\mathbf{V}^{(i)}$

$$\mathbf{V}^{(i)} \leftarrow \mathbf{V}^{(i)} \odot \sqrt{\frac{\mathbf{X}^{(i)^T}\mathbf{U}^{(i)} + \alpha\mathbf{A}_v^{(i)}\mathbf{V}^{(i)}}{((\mathbf{R}^{(i)^T}+w^2\mathbf{V}^{(i)}\mathbf{U}^{(i)^T})\mathbf{U}^{(i)} + \alpha\mathbf{D}_v^{(i)}\mathbf{V}^{(i)} + 0.5\gamma}} \tag{5}$$

Based on Eq (3)-(5), we develop the iterative multiplicative updating algorithm for optimization as described in our main paper.

Convergence analysis

We use the auxiliary function approach [2] to prove the convergence of updating rules. We first introduce the definition of the auxiliary function.

Definition 1 *A function $Z(h,\hat{h})$ is an auxiliary function for a given function $J(h)$ if the conditions $Z(h,\hat{h}) \geq J(h)$ and $Z(h,h) = J(h)$ are satisfied for any given $h, \hat{h}$ [2].*

Lemma 1 *If Z is an auxiliary function for J, then J is non-increasing under the update $h^{(t+1)} = \underset{h}{\operatorname{argmin}} Z(h, h^{(t)})$ [2].*

Next, we show that updating $\mathbf{U}^{(1)}$ according to Eq (3) will monotonically decrease the objective function[1] until convergence by the following theorem.

Theorem 1 *let $L(\mathbf{U}^{(1)})$ denote the sum of all terms in the objective function as Eq. (1) that contain $\mathbf{U}^{(1)}$, then the following function*

$$
\begin{aligned}
Z(\mathbf{U}^{(1)}, \widetilde{\mathbf{U}}^{(1)}) = &-2\sum_{pq}[(\mathbf{W}^{(1)} \odot \mathbf{W}^{(1)} \odot \mathbf{X}^{(1)})\mathbf{V}^{(1)}]_{pq}\widetilde{\mathbf{U}}^{(1)}_{pq}(1+\log\frac{\mathbf{U}^{(1)}_{pq}}{\widetilde{\mathbf{U}}^{(1)}_{pq}}) \\
&+\sum_{pq}\frac{[(\mathbf{W}^{(1)} \odot \mathbf{W}^{(1)} \odot (\widetilde{\mathbf{U}}^{(1)}{\mathbf{V}^{(1)}}^T))\mathbf{V}^{(1)}]_{pq}(\mathbf{U}^{(1)}_{pq})^2}{\widetilde{\mathbf{U}}^{(1)}_{pq}} \\
&+\sum_{pq}\frac{[\alpha\mathbf{D}^{(1)}_u\widetilde{\mathbf{U}}^{(1)}]_{pq}(\mathbf{U}^{(1)}_{pq})^2}{\widetilde{\mathbf{U}}^{(1)}_{pq}} - \sum_{pqr}\alpha(\mathbf{A}^{(1)}_u)_{pr}\widetilde{\mathbf{U}}^{(1)}_{rq}\widetilde{\mathbf{U}}^{(1)}_{pq}\mathbf{\Psi}(p,r,q) + \sum_{pq}\beta\mathbf{\Gamma}_{pq}\frac{(\mathbf{U}^{(1)}_{pq})^4}{(\widetilde{\mathbf{U}}^{(1)}_{pq})^3} \\
&-2\sum_{pqr}[\beta{\mathbf{S}^{(1,2)}}^T\mathbf{S}^{(1,2)}\widetilde{\mathbf{U}}^{(1)}(\widetilde{\mathbf{U}}^{(1)})^T{\mathbf{S}^{(1,2)}}^T]_{rp}\mathbf{\Psi}(p,r,q) + \frac{\gamma}{4}\sum_{pq}\frac{(\mathbf{U}^{(1)}_{pq})^4 + 3(\widetilde{\mathbf{U}}^{(1)}_{pq})^4}{(\widetilde{\mathbf{U}}^{(1)}_{pq})^3}
\end{aligned}
\tag{6}
$$

is an auxiliary function for $L(\mathbf{U}^{(1)})$. where $\mathbf{\Psi}(p,r,q) = 1 + \log\frac{\mathbf{U}^{(1)}_{rq}\mathbf{U}^{(1)}_{pq}}{\widetilde{\mathbf{U}}^{(1)}_{rq}\widetilde{\mathbf{U}}^{(1)}_{pq}}$ and $\mathbf{\Gamma} = (\widetilde{\mathbf{U}}^{(1)})^T{\mathbf{S}^{(1,2)}}^T\mathbf{S}^{(1,2)}\widetilde{\mathbf{U}}^{(1)}(\widetilde{\mathbf{U}}^{(1)})^T{\mathbf{S}^{(1,2)}}^T\mathbf{S}^{(1,2)}$. Furthermore, it is a convex function w.r.t. $\mathbf{U}^{(1)}$ and has a global minimum.

Theorem 1 can be proofed by using a similar idea to that in the work [3] by validating (1) $Z(\mathbf{U}^{(1)}, \widetilde{\mathbf{U}}^{(1)}) \geq L(\mathbf{U}^{(1)})$. (2) $Z(\mathbf{U}^{(1)}, \mathbf{U}^{(1)}) = L(\mathbf{U}^{(1)})$ (3) the Hessian matrix $\nabla^2_{\mathbf{U}^{(1)}}$ is positive definite. We thus omit the details.

The convergence proof of updating equation for $\mathbf{U}^{(2)}$ and $\mathbf{V}^{(i)}$ (for $i = 1,2$) can be proven with a similar strategy.

References

1. Boyd, Stephen, and Lieven Vandenberghe. Convex optimization. Cambridge university press, 2004.

2. Lee, Daniel D., and H. Sebastian Seung. Algorithms for non-negative matrix factorization." Advances in neural information processing systems. In Advances in neural information processing systems, pp. 556-562. 2001.

3. Ding, Chris, Tao Li, Wei Peng, and Haesun Park. Orthogonal nonnegative matrix t-factorizations for clustering. In Proceedings of the 12th ACM SIGKDD international conference on Knowledge discovery and data mining, pp. 126-135. ACM, 2006.

[1] The objective function (3) in the main paper.
