# Supplementary figures and images for: iDrug: Integration of drug repositioning and drug-target prediction via cross-network embedding
Source: PLoS Comput Biol. 2020 Jul 15;16(7):e1008040. doi: 10.1371/journal.pcbi.1008040 (PMC7384678; doi:10.1371/journal.pcbi.1008040)

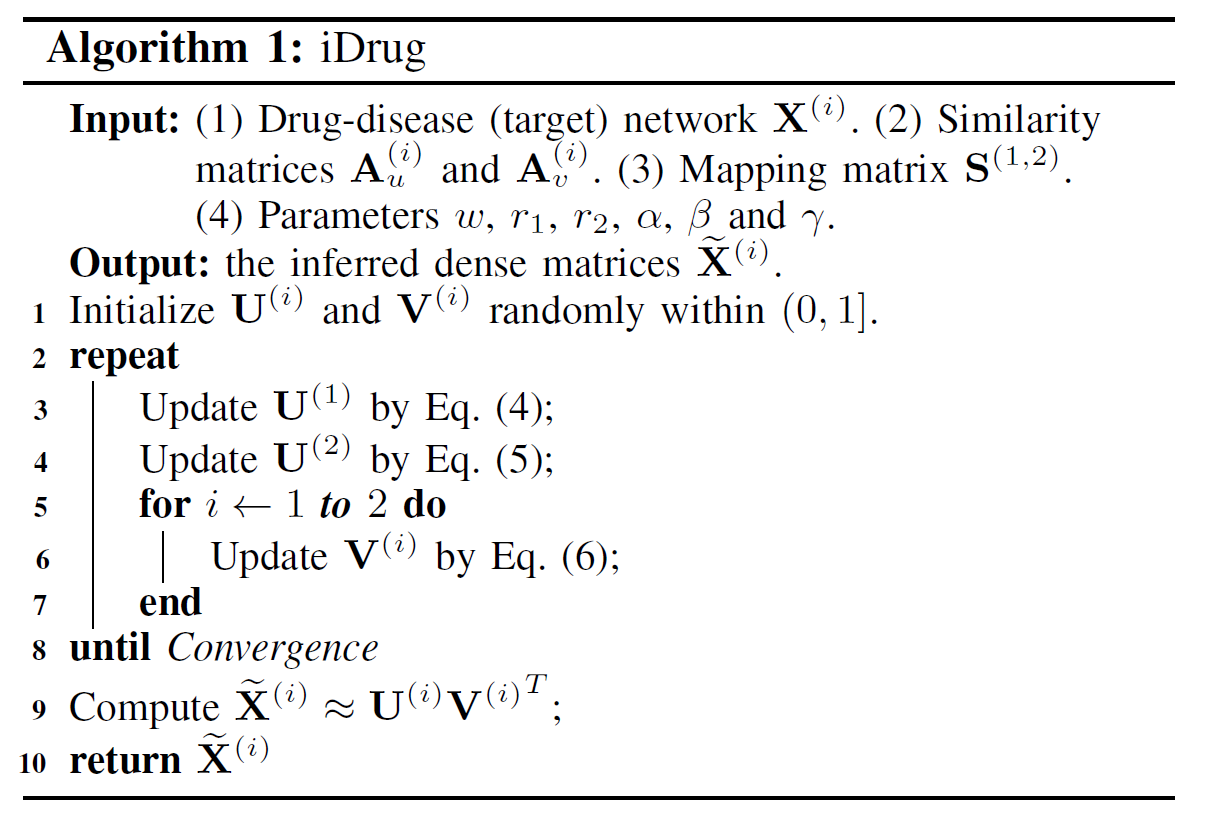

Supplement: S1 Fig — The algorithm to solve the objective function in Eq (3). (TIF) [file pcbi.1008040.s002.tif]

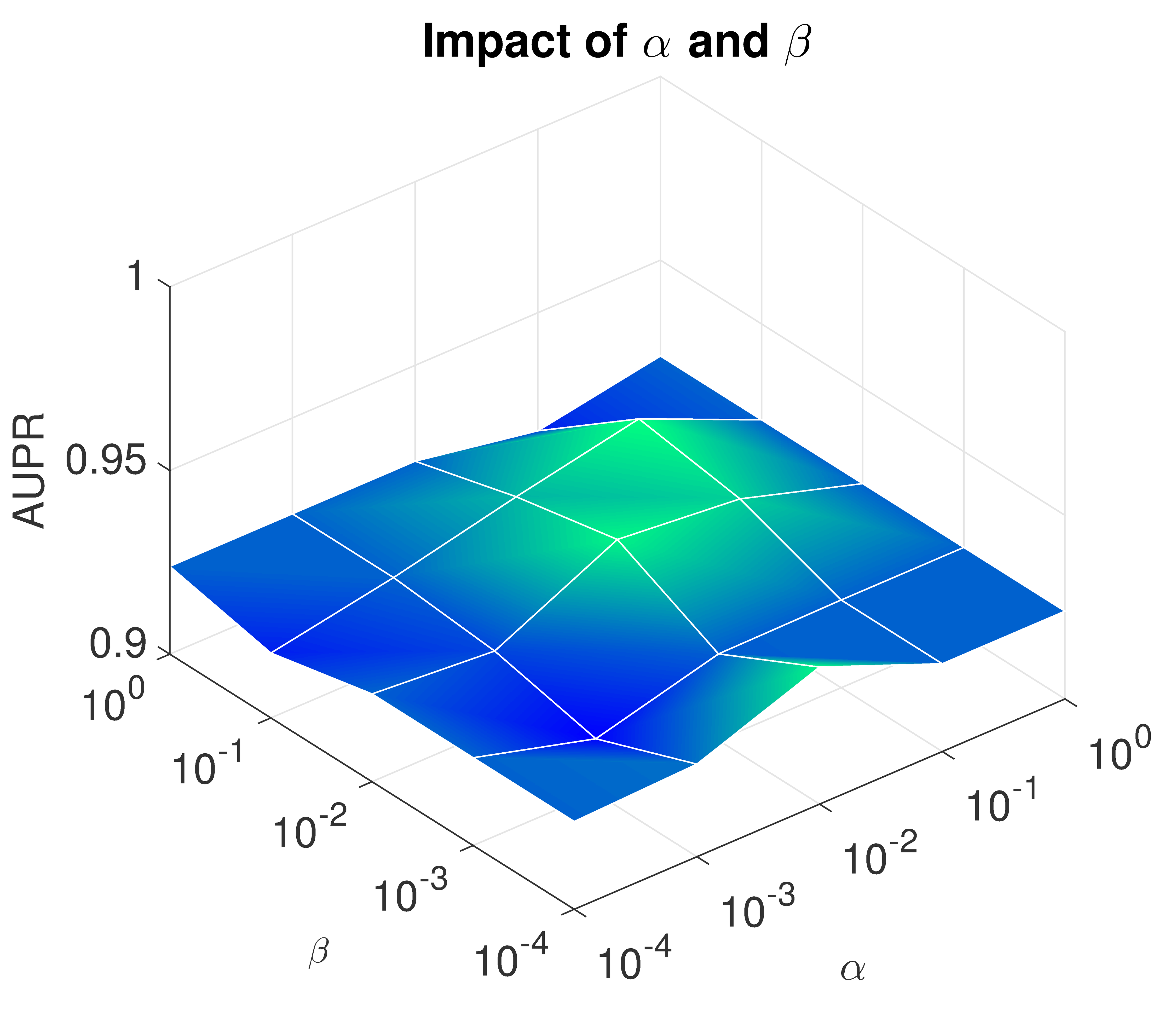

Supplement: S2 Fig — Grid-based search method to study the impact of α and β with respect to the AUPR measurement for the task of drug repositioning, while γ is fixed to be 0.01. (TIF) [file pcbi.1008040.s003.tif]

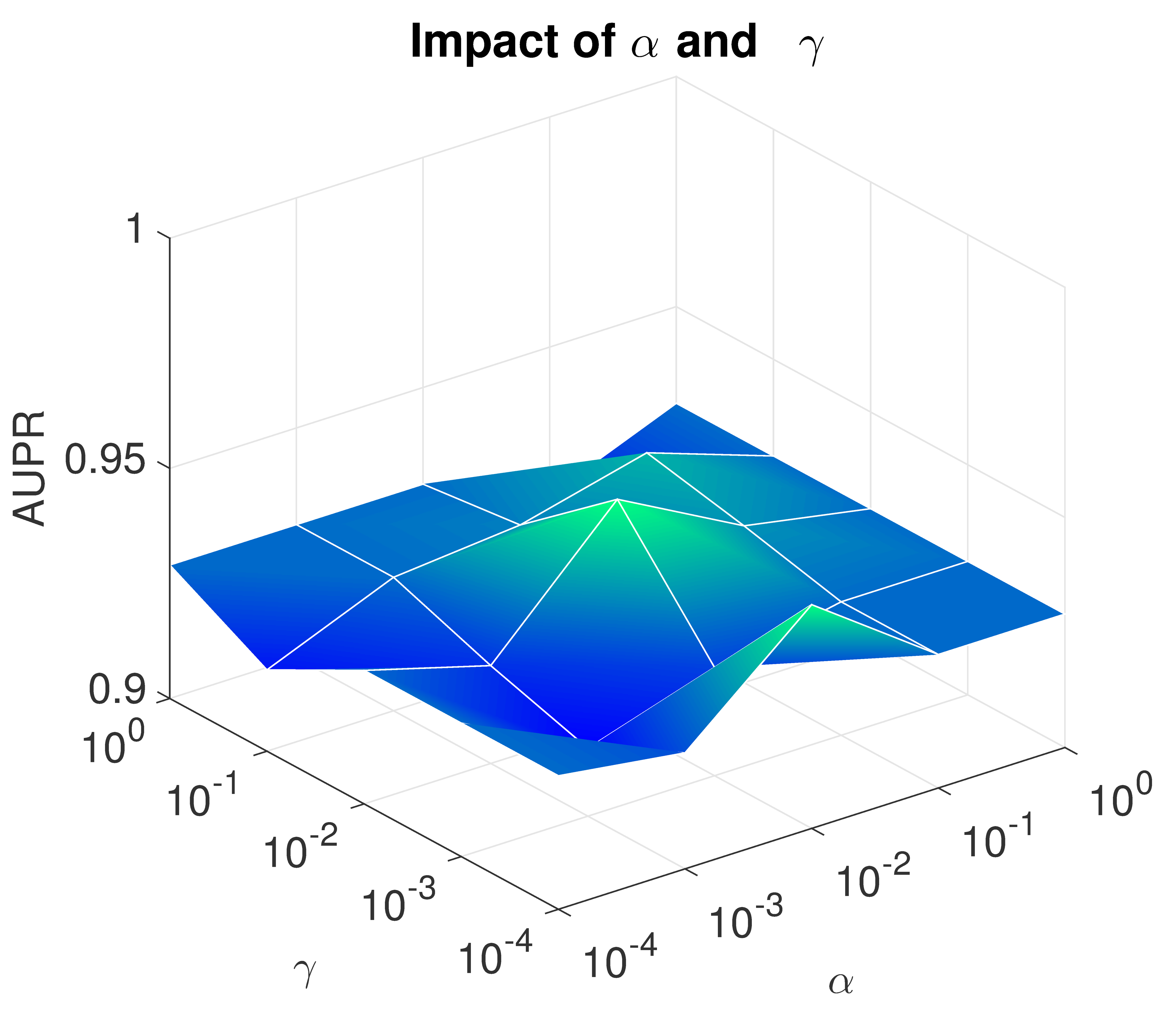

Supplement: S3 Fig — Grid-based search method to study the impact of α and γ with respect to the AUPR measurement for the task of drug repositioning, while β is fixed to be 0.01. (TIF) [file pcbi.1008040.s004.tif]

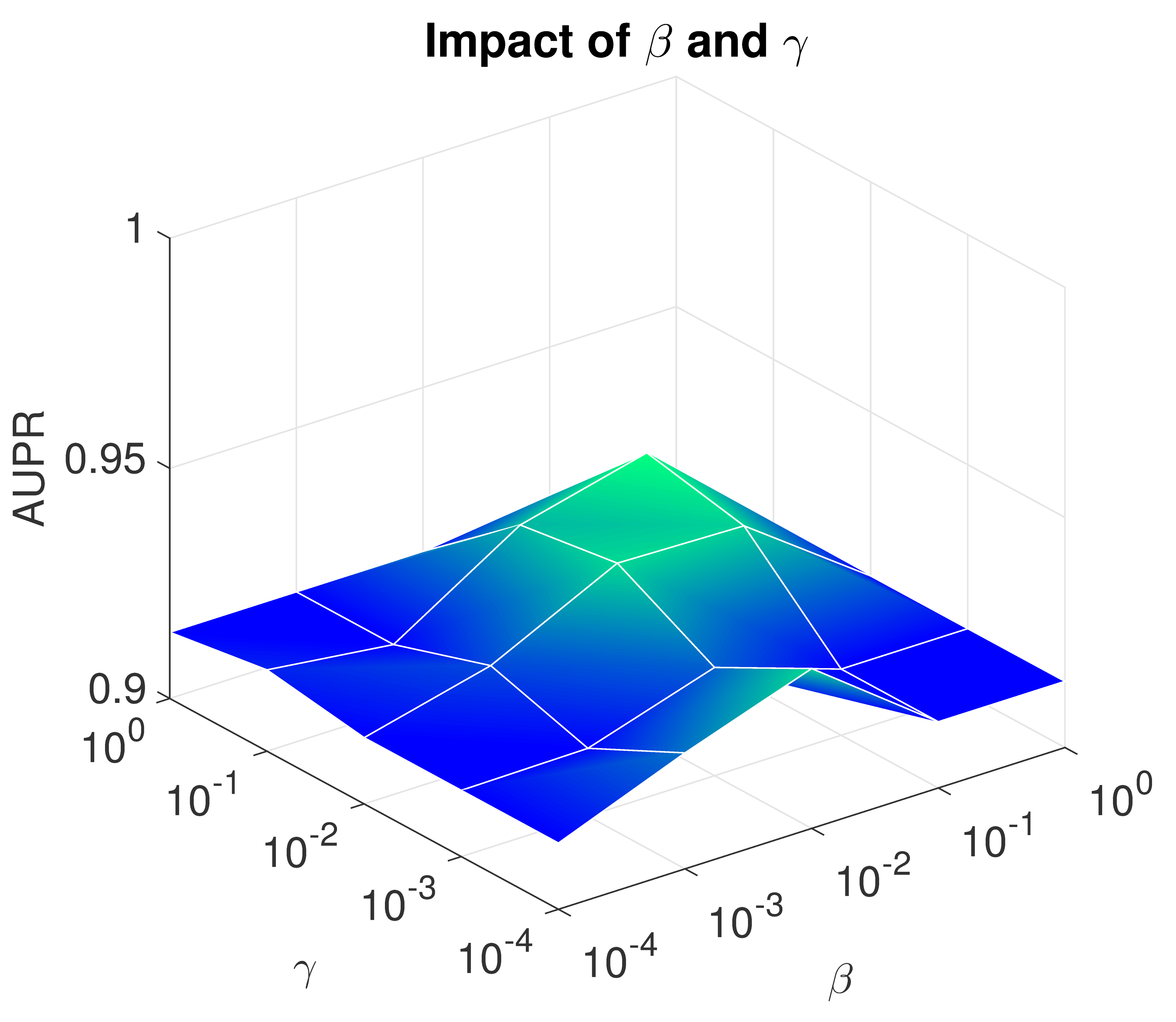

Supplement: S4 Fig — Grid-based search method to study the impact of β and γ with respect to the AUPR measurement for the task of drug repositioning, while α is fixed to be 0.01. (TIF) [file pcbi.1008040.s005.tif]

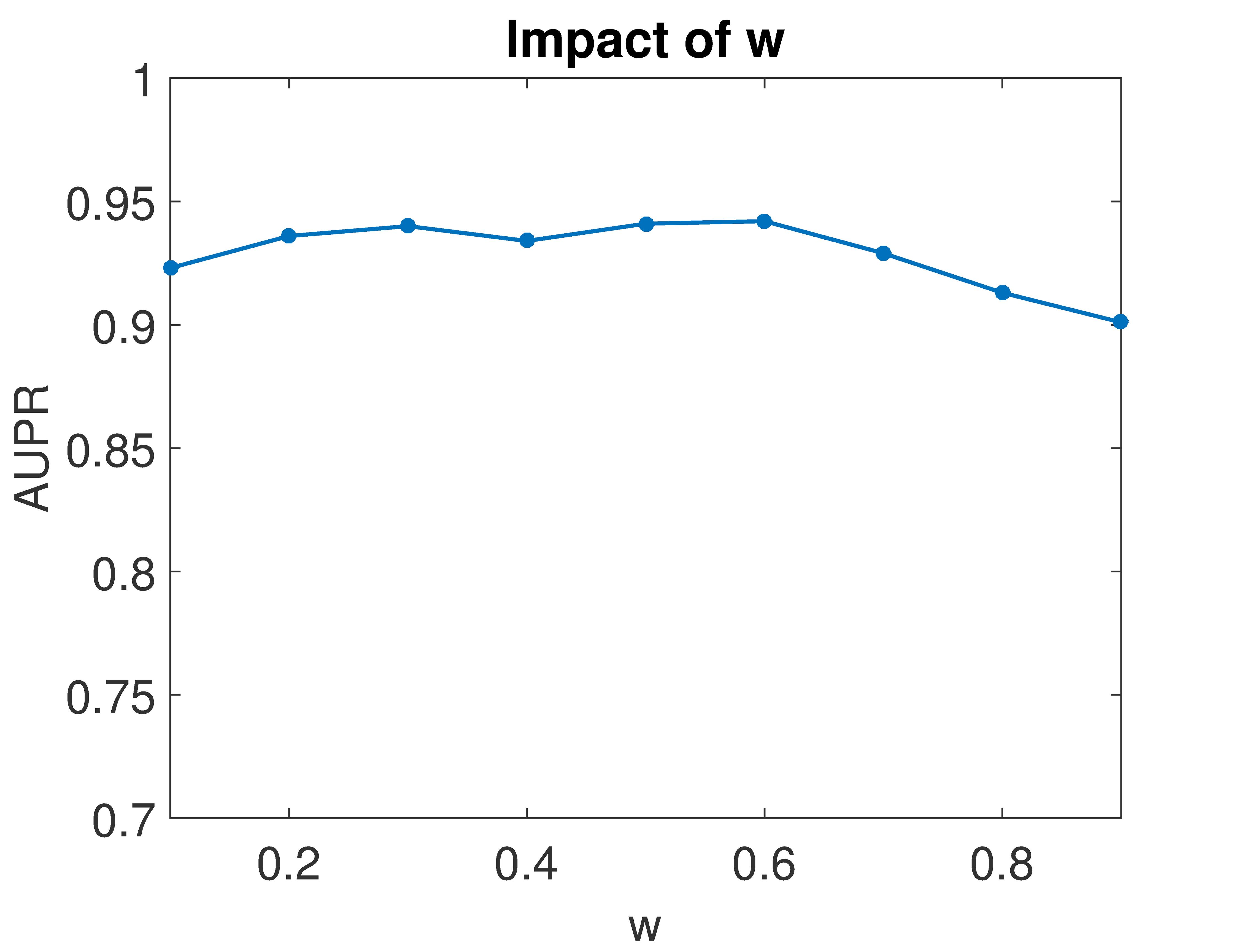

Supplement: S5 Fig — The impact of w with respect to the AUPR measurement for the task of drug repositioning. (TIF) [file pcbi.1008040.s006.tif]

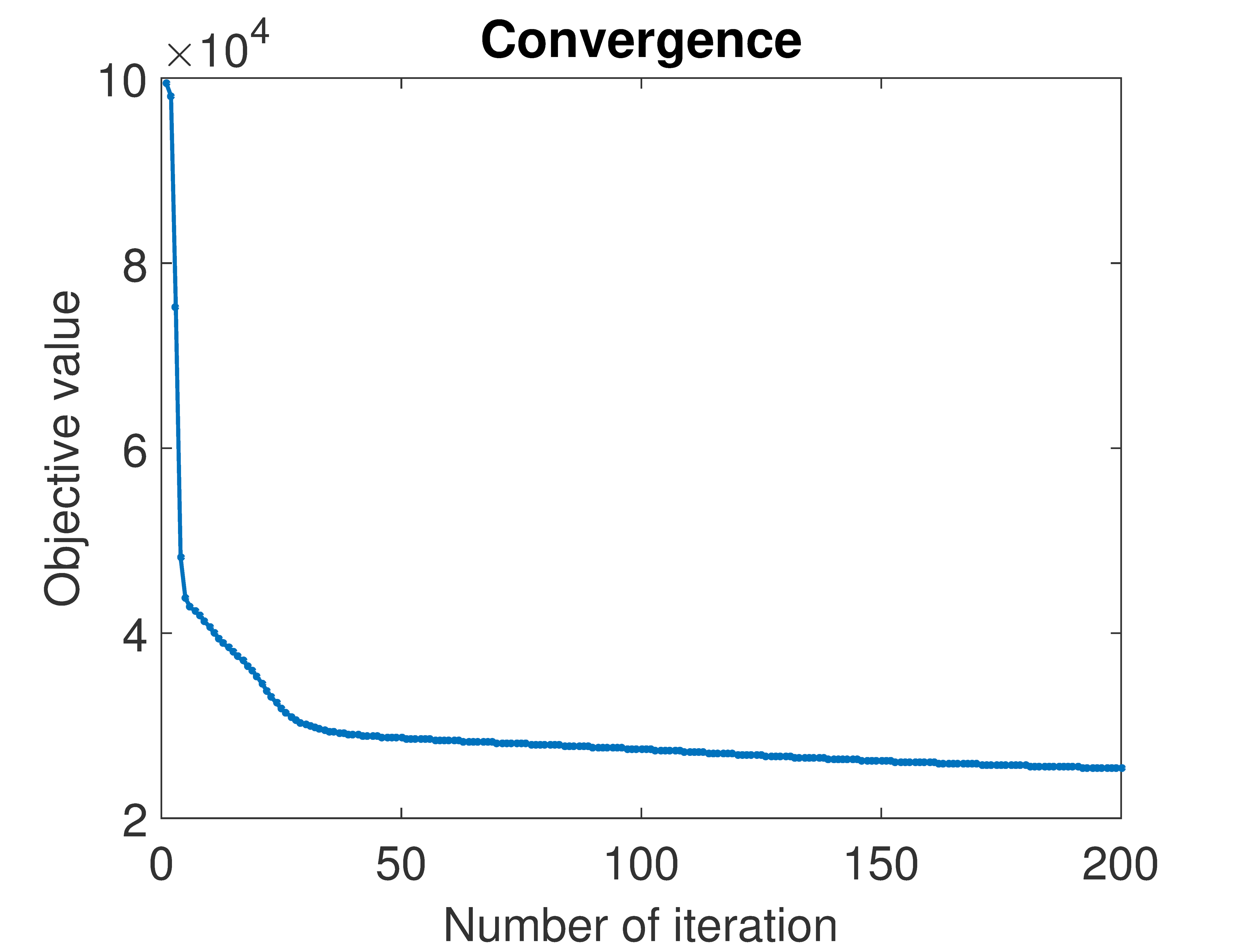

Supplement: S6 Fig — The convergence of iDrug on empirical data for the task of drug repositioning. (TIF) [file pcbi.1008040.s007.tif]
